# Supplementary material for: Phylogenetic Assignment of the Fungicolous Hypoxylon invadens (Ascomycota, Xylariales) and Investigation of its Secondary Metabolites
Source: Microorganisms. 2020 Sep 11;8(9):1397. doi: 10.3390/microorganisms8091397 (PMC7565716; doi:10.3390/microorganisms8091397)
Supplement: Supplementary file 1 [file microorganisms-08-01397-s001.zip › microorganisms-892779 supple and non published/microorganisms-892779 proofed non published materials.docx]

Article

Phylogenetic Assignment of the Fungicolous *Hypoxylon invadens* (Ascomycota, Xylariales) and Investigation of its Secondary Metabolites

Kevin Becker ^1,2^, Christopher Lambert ^1,2,3^, Jörg Wieschhaus ^1^ and Marc Stadler ^1,2,^*

^1^ Department of Microbial Drugs, Helmholtz Centre for Infection Research GmbH (HZI), Inhoffenstraße 7, 38124 Braunschweig, Germany; [kevin.becker@helmholtz-hzi.de](mailto:kevin.becker@helmholtz-hzi.de) (K.B.), [christopher.lambert@helmholtz‑hzi.de](mailto:christopher.lambert@helmholtzhzi.de) (C.L.), [joerg.wieschhaus@gmail.com](mailto:joerg.wieschhaus@gmail.com) (J.W.)

^2^ German Centre for Infection Research Association (DZIF), Partner site Hannover-Braunschweig, Inhoffenstraße 7, 38124 Braunschweig, Germany

^3^ Department for Molecular Cell Biology, Helmholtz Centre for Infection Research GmbH (HZI) Inhoffenstraße 7, 38124 Braunschweig, Germany

***** Correspondence: [marc.stadler@helmholtz-hzi.de](mailto:marc.stadler@helmholtz-hzi.de) (M.S.); Tel.: +49-531-6181-4240; Fax: +49-531-6181-9499

Received: 23 July 2020; Accepted: date; Published: date

**Abstract:** The ascomycete *Hypoxylon invadens* was described in 2014 as a fungicolous species growing on a member of its own genus, *H.* *fragiforme*, which is considered a rare lifestyle in the Hypoxylaceae. This renders *H.* *invadens* an interesting target in our efforts to find new bioactive secondary metabolites from members of the Xylariales. So far, only volatile organic compounds have been reported from *H.* *invadens*, but no investigation of non-volatile compounds had been conducted. Furthermore, a phylogenetic assignment following recent trends in fungal taxonomy via a multiple sequence alignment seemed practical. A culture of *H.* *invadens* was thus subjected to submerged cultivation to investigate the produced secondary metabolites, followed by isolation via preparative chromatography and subsequent structure elucidation by means of Nuclear Magnetic Resonance (NMR) Spectroscopy and High-Resolution Mass Spectrometry (HR‑MS). This approach led to the identification of the known flaviolin (**1**) and 3,3‑biflaviolin (**2**) as main components, which had never been reported from the order Xylariales before. Assessment of their antimicrobial or cytotoxic effects via a panel of commonly used microorganisms and cell lines in our laboratory did not yield effects of relevance. Concurrently, genomic DNA from the fungus was used to construct a multigene phylogeny using ribosomal sequence information from the internal transcribed spacer region (ITS), the 28S large subunit of ribosomal DNA (LSU), and proteinogenic nucleotide sequences from the second largest subunit of the DNA-directed RNA polymerase II (RPB2), and *β*-tubulin (TUB2) genes. A placement in a newly formed clade with *H.* *trugodes* was strongly supported in a Maximum-Likelihood (ML) phylogeny using sequences derived from well characterized strains, but the exact position of said clade remains unclear. Both, the chemical and the phylogenetic results suggest further inquiries into the lifestyle of this unique fungus to get a better understanding of both its ecological role and function of its produced secondary metabolites hitherto unique to the Xylariales.

**Keywords:** Ascomycota; chromatography; multigene phylogeny; naphthoquinones; taxonomy; Xylariales

1. Introduction

*Hypoxylon* is the largest genus within the fungal family Hypoxylaceae (order Xylariales, Ascomycota) with more than 230 described species [1]. The stromata (fruiting bodies) of *Hypoxylon* are often rather conspicuous and can frequently be found on dead wood in all forested areas of the world, but the mycelia of these fungi are also among the most frequently isolated endophytes of seed plants. This underlines the ecological importance of the genus [2,3]. Recent taxonomical advances revolved on the reorganization of *Hypoxylon* and allies, using a polyphasic concept combining morphological, molecular phylogenetics with special emphasis on multi-locus genealogies and chemotaxonomic approaches. This ultimately led to the resurrection of the Hypoxylaceae itself and the segregation of genera like *Hypomontagnella* and *Jackrogersella* [2,3], while increasing chemotaxonomical knowledge supported the treatment of genera like *Annulohypoxylon* as an own genus where sequence information derived from the widely used Internal transcribed spacer region (ITS) alone did not give a conclusive resolution [4,5]. Members of the Hypoxylacaeae are also known to produce an astonishing amount of diverse secondary metabolites, making them valuable targets for natural product researchers [6]. This research branch is further supported by the increasing number of high-quality genomes available [7], expanding the classical screening approaches of natural product chemistry by the possibility for genome mining and biosynthesis studies of promising bioactive compounds.

The monotypic species *Hypoxylon invadens* was recently reported from France as a fungicolous species growing on stromata of a member of its own genus, *H. fragiforme* [8]. Only a few cases of this apparently mycophilic lifestyle are known from the genus *Hypoxylon* and other genera that now belong to the Hypoxylaceae (i.e., *Chlorostroma* and *Thuemenella* [9], of which no mycelial cultures of these fungi are presently available in the public domain), and it was not possible to evaluate whether the stromata just grow coincidentally on the stromata of their relatives, or there is some form of parasitism or mutualism involved. We were able to obtain a culture from the ascospores of the holotype specimen of *H.* *invadens* that can now be studied in-depth for its physiological and ecological traits, including secondary metabolite production. In a previous study we have checked the cultures of *H.* *invadens* and *H. macrocarpum* [8], and the only reported metabolites from those species are several volatile organic compounds (VOC) [10,11], which were investigated by GC-MS analysis and total synthesis after observation of a strong odor originating from mycelia grown on oatmeal agar [8].

The current study is dedicated to assess the phylogenetic affinities of *H.* *invadens* via a four-locus phylogeny, as well as the investigation of its secondary metabolites, followed by evaluation of the cytotoxicity and antimicrobial activity of the newly isolated compounds.

2. Materials and Methods

2.1. General

Electrospray Mass (ESI-MS) spectra were recorded with an UltiMate^®^ 3000 Series UHPLC (Thermofisher Scientific, Waltman, MA, USA) utilizing a C18 Acquity^®^ UPLC BEH column (2.1×50 mm, 1.7 µm; Waters, Milford, MA, USA), connected to an amaZon speed^®^ ESI‑Iontrap‑MS (Bruker, Billerica, MA, USA). HPLC parameters were set as follows: solvent A: H_2_O+0.1% formic acid, solvent B: acetonitrile (ACN)+0.1% formic acid; gradient: 5% B for 0.5 min, increasing to 100% B in 19.5 min, keeping 100% B for further 5 min; flowrate 0.6 mL/min, DAD detection 200−600 nm.

High Resolution Electrospray Mass (HR‑ESI‑MS) spectra were obtained with an Agilent 1200 Infinity Series HPLC (Agilent Technologies, Santa Clara, CA, USA) connected to a maXis^®^ Electrospray Time-of-flight Mass Spectrometer (ESI‑TOF‑MS; Bruker; HPLC conditions same as for ESI-MS measurements).

Nuclear Magnetic Resonance (NMR) spectra were recorded with an Avance III 500 spectrometer (Bruker, ^1^H NMR: 500 MHz, ^13^C NMR: 125 MHz). UV/vis spectra were taken with a spectrophotometer UV-2450 (Shimadzu, Kyoto, Japan).

**Table 1.** Sequence accession numbers, corresponding isolates and literature reference of sequences used for the molecular phylogenetic inference. Species-type specimen are given the corresponding label (HT = holotype; ET = Epitype). The newly sequenced *H. invadens* isolate is highlighted in bold.

| **Species** | **Status** | **Strain** | **Origin** | **GenBank Acc. No. of DNA loci** | | | | **References** |
| --- | --- | --- | --- | --- | --- | --- | --- | --- |
|  |  |  |  | **ITS** | **LSU** | **RPB2** | **TUB2** |  |
| *Annulohypoxylon annulatum* | ET | CBS 140775 | Texas | KY610418 | KY610418 | KY624263 | KX376353 | [2,12] |
| *A. annulatum* |  | DSM 107931 | Texas | MK287534  AJ390397 | MK287546 | MK287559 | MK287572 | [13] |
| *A. moriforme* |  | CBS 123579 | Martinique | KX376321 | KY610425 | KY624289 | KX271261 | [2,14] |
| *A. nitens* |  | MFLUCC 12.0832 | Thailand | KJ934991 | KJ934992 | KJ934994 | KJ934993 | [15] |
| *A. stygium* |  | MUCL 54601 | French Guinea | KY610409 | KY610475 | KY624292 | KX271263 | [2] |
| *A. truncatum* | ET | CBS 140778 | Texas | KY610419 | KY610419 | KY624277 | KX376352 | [2,12] |
| *A. truncatum* |  | DSM 107925 | Texas | MK287531 | MK287543 | MK287556 | MK287569 | [13] |
| *Daldinia concentrica* |  | CBS 113277 | Germany | AY616683 | KY610434 | KY624243 | KC977274 | [2,16,17] |
| *D. eschscholtzii* |  | MUCL 45435 | Benin | JX658484 | KY610437 | KY624246 | KC977266 | [2,16,18] |
| *D. petriniae* | ET | MUCL 49214 | Austria | AM749937 | KY610439 | KY624248 | KC977261 | [2,16,19] |
| *D. placentiformis* |  | MUCL 47603 | Mexico | AM749921 | KY610440 | KY624249 | KC977278 | [2,16,19] |
| *Entonaema liquescens* |  | ATCC 46302 | USA | KY610389 | KY610443 | KY624253 | KX271248 | [2] |
| *Graphostroma platystoma* | HT | CBS 270.87 | France | JX658535 | DQ836906 | KY624296 | HG934108 | [2,18,20,21] |
| *Hypom. barbarensis* | HT | STMA 14081 | Argentina | MK131720 | MK131718 | MK135891 | MK135893 | [3] |
| *Hypom. monticulosa* | ET | MUCL 54604 | French Guiana | KY610404 | KY610487 | KY624305 | KX271273 | [2] |
| *Hypom. submonticulosa* |  | CBS 115280 | France | KC968923 | KY610457 | KY624226 | KC977267 | [2,16] |
| *Hypoxylon carneum* |  | MUCL 54177 | France | KY610400 | KY610480 | KY624297 | KX271270 | [2] |
| *H. cercidicola* |  | CBS 119009 | France | KC968908 | KY610444 | KY624254 | KU684189 | [2,16] |
| *H. croceum* |  | DSM 107926 | Texas | MK287535 | MK287547 | MK287560 | MK287573 | [13] |
| *H. croceum* |  | DSM 107932 | Texas | MK287532 | MK287544 | MK287557 | MK287570 | [13] |
| *H. crocopeplum* |  | CBS 119004 | France | KC968907 | KY610445 | KY624255 | KC977268 | [2,16] |
| *H. fendleri* |  | MUCL 54792 | French Guinea | KF234421 | KY610481 | KY624298 | KF300547 | [2,16] |
| *H. fragiforme* | ET | MUCL 51264 | Germany | KC477229 | KM186295 | MK887342 | KX271282 | [2,13,15,22] |
| *H. fuscum* | ET | CBS 113049 | France | KY610401 | KY610482 | KY624299 | KX271271 | [2] |
| *H. griseobrunneum* | T | CBS 331.73 | India | KY610402 | KY610483 | KY624300 | KC977303 | [2,16] |
| *H. guilanense* | HT | MUCL 57726 | Iran | MT214997 | MT214992 | MT212235 | MT212239 | [23] |
| *H. haematostroma* | ET | MUCL 53301 | Martinique | KC968911 | KY610484 | KY624301 | KC977291 | [2,16] |
| *H. howeanum* |  | MUCL 47599 | Germany | AM749928 | KY610448 | KY624258 | KC977277 | [2,16,19] |
| *H. hypomiltum* |  | MUCL 51845 | Guadeloupe | KY610403 | KY610449 | KY624302 | KX271249 | [2] |
| ***H. invadens*** | **HT** | **MUCL 51475** | **France** | **MT809133** | **MT809132** | **MT813037** | **MT813038** | **This Study** |
| *H. investiens* |  | CBS 118183 | Malaysia | KC968925 | KY610450 | KY624259 | KC977270 | [2,16] |
| *H. lateripigmentum* | HT | MUCL 53304 | Martinique | KC968933 | KY610486 | KY624304 | KC977290 | [2,16] |
| *H. lenormandii* |  | CBS 119003 | Ecuador | KC968943 | KY610452 | KY624261 | KC977273 | [2,16] |
| *H. lienhwacheense* |  | MFLUCC 14-1231 | Thailand | KU604558 | MK287550 | MK287563 | KU159522 | [13,24] |
| *H. musceum* |  | MUCL 53765 | Guadeloupe | KC968926 | KY610488 | KY624306 | KC977280 | [2,16] |
| *H. olivaceopigmentum* | T | DSM 107924 | Texas | MK287530 | MK287542 | MK287555 | MK287568 | [13] |
| *H. papillatum* | HT | ATCC 58729 | USA | KC968919 | KY610454 | KY624223 | KC977258 | [2,16] |
| *H. perforatum* |  | CBS 115281 | France | KY610391 | KY610455 | KY624224 | KX271250 | [2] |
| *H. petriniae* | HT | CBS 114746 | France | KY610405 | KY610491 | KY624279 | KX271274 | [2,12] |
| *H. pilgerianum* |  | STMA 13455 | Martinique | KY610412 | KY610412 | KY624308 | KY624315 | [2] |
| *H. porphyreum* |  | CBS 119022 | France | KC968921 | KY610456 | KY624225 | KC977264 | [2,25] |
| *H. pulicicidum* | HT | CBS 122622 | Martinique | JX183075 | KY610492 | KY624280 | JX183072 | [2,26] |
| *H. rickii* | ET | MUCL 53309 | Martinique | KC968932 | KY610416 | KY624281 | KC977288 | [2,16] |
| *H. rubiginosum* | ET | MUCL 52887 | Germany | KC477232 | KY610469 | KY624266 | KY624311 | [2,22] |
| *H. samuelsii* | ET | MUCL 51843 | Guadeloupe | KC968916 | KY610466 | KY624269 | KC977286 | [2,16] |
| *H. texense* | T | DSM 107933 | Texas | MK287536 | MK287548 | MK287561 | MK287574 | [13] |
| *H. ticinense* |  | CBS 115273 | France | JQ009317 | KY610471 | KY624272 | AY951757 | [2,4] |
| *H. trugodes* | ET | MUCL 54794 | Sri Lanka | KF234422 | KY610493 | KY624282 | KF300548 | [2,16] |
| *H. vogesiacum* |  | CBS 115273 | France | KC968920 | KY610417 | KY624283 | KX271275 | [2,16] |
| *Jackrogersella cohaerens* |  | CBS 119126 | Germany | KY610396 | KY610497 | KY624270 | KY624314 | [2] |
| *J. minutella* |  | CBS 119015 | Portugal | KY610381 | KY610424 | KY624235 | KX271240 | [2] |
| *J. multiformis* | ET | CBS 119016 | Germany | KC477234 | KY610473 | KY624290 | KX271262 | [2,12,16] |
| *Pyrenopolyporus hunteri* | ET | MUCL 52673 | Ivory Coast | KY610421 | KY610472 | KY624309 | KU159530 | [2,12] |
| *P. laminosus* | HT | MUCL 53305 | Martinique | KC968934 | KY610485 | KY624303 | KC977292 | [2,16] |
| *P.nicaraguensis* |  | CBS 117739 | Burkina Faso | AM749922 | KY610489 | KY624307 | KC977272 | [2,16,19] |
| *Rhopalostroma angolense* |  | CBS 126414 | Ivory Coast | KY610420 | KY610459 | KY624228 | KX271277 | [2] |
| *Thamnomyces dendroidea* | HT | CBS 123578 | French Guinea | FN428831 | KY610467 | KY624232 | KY624313 | [2,27] |
| *Xylaria hypoxylon* | ET | CBS 122620 | Sweden | KY610407 | KY610495 | KY624231 | KX271279 | [2,28] |

2.2. Fungal Material and Molecular Phylogeny

A culture from the holotype specimen of *Hypoxylon invadens* was used in this study. A well-grown culture of *H.* *invadens* on YM 6.3 agar was used for genomic DNA (gDNA) extraction using a commercially available kit as described by Kuhnert et al. [16]. The obtained gDNA was used to amplify four different loci: the internal transcribed spacer region (ITS), the 28S large subunit of ribosomal DNA (LSU), the second largest subunit of the DNA-directed RNA polymerase II (RPB2) and *β*-tubulin (TUB2) with primers as described elsewhere [29–33]. PCR programs, subsequent amplicon purification, and sequencing were conducted as described previously [9,34].

Sequence assembly and analysis was done following Wendt et al. [2] with the program package Geneious^®^ 7.1.9 (<http://www.geneious.com>, [35]). Taxon selection and complementation of sequences was based on the dataset of Sir et al. [13], which comprised many type strains as well as authentic representatives of all phylogenetic lineages of the genus *Hypoxylon* and allied Hypoxylaceae. The GenBank accession numbers of the sequences used are given in Table 1. Loci were aligned using the in-built version of the program MAFFT 7.017 with settings for the G-INS-I algorithm set to default [36]. The resulting alignment was filtered for phylogenetic informative positions via the server version of Gblocks (Castresana Labs, [37]); low stringency settings with options for smaller final blocks and gap positions within final blocks, see Talavera and Castresana [38] and Wendt et al. [2]. Molecular phylogenetic inference followed using the client version of IQTree (Version 2.0-RC2, [39]) with a partitioned dataset [40] and ModelFinder for model selection [41]. Non-parametric bootstrap (BS, **≥**50%, majority consensus [42]) and Shimodaira-Hasegawa approximate Likelihood-Ratio-Test (SH‑aLRT, ≥85% [43,44]) values were assigned to the best scoring Maximum-Likelihood (ML) tree.

2.3. Cultivation, Extraction, and Isolation

For cultivation*,* seed cultures were prepared by adding three mycelial plugs of ca. 0.25 mm² size each from well-grown yeast-malt agar plates (YM 6.3 [45], supplemented with 20 g/L agar) into 250 mL shaking flasks containing 100 mL of liquid YM 6.3 medium*.* Seed cultures were inoculated on a rotary shaker *(*Infors, Bottmingen, Switzerland) for 13 d (23 °C, 140 rpm). For preparation of the submerged cultures, 20 × 500 mL shaking flasks containing 200 mL of ZM‑½ medium [45] were inoculated with 3 mL of seed culture. The flasks were incubated on a rotary shaker under the aforementioned conditions and consumption of glucose was monitored by using glucose test stripes (Macherey-Nagel, Düren, Germany) daily. Besides glucose, a visual estimation of the red colour of the culture broth was taken into account to determine the termination of fermentation. Accordingly, three days after the glucose test stripes indicated that the glucose had been consumed (and the colour was of an intense red), the cultures were harvested and extracted.

After termination of fermentations, supernatant and mycelium were separated by centrifugation and subsequent gauze filtration. The mycelium was extracted two times, first using 1 L of ethyl acetate (EtOAc) and then 1 L of acetone as organic phases at 40 °C in an ultra-sonic bath for one hour. Both organic phases were individually extracted twice with ca. 1 L of deionized water using a separatory funnel and the aqueous phases discarded.

The organic phases of the two extractions of the ZM‑½ medium were combined and dried *in vacuo* to yield the crude extract (526 mg). An aliquot of this extract was dissolved in DMSO and subjected to HPLC-DAD/MS analysis (see Figure S1).

The crude extract of the culture prepared in ZM‑½ medium was gradually separated using two subsequent steps of chromatography to yield the pure compounds **1** and **2**. As a first step, the crude extract was dissolved in 4 mL ACN:H_2_O (75:25) and transferred to an open solid phase cartridge (Strata^®^ X 33 µm Polymeric Reversed Phase Tube, 1 g/12 mL, SN 8B‑S100‑JDG; Phenomenex, Aschaffenburg, Germany). Elution was achieved by using low vacuum (*ca.* 800 mbar) and a step-gradient of H_2_O:ACN (90:10, 60:40, 30:70, 100:0; 4 mL per step). The effluents of the gradient steps 90:10 and 60:40 were combined according to ESI‑MS results and dried *in vacuo*, yielding 221 mg of extract.

The resulting crude extract was further separated by dissolution in ca. 5 mL of ACN:H_2_O (60:40) and subjection to a PLC 2250 HPLC system (Gilson, Middleton, WI, USA), utilizing a Nucleodur^®^ C18ec column (125 × 40 mm, 7 µm; Macherey-Nagel, Düren, Germany) and solvents: A: H_2_O+0.1% formic acid; B: ACN+0.1% formic acid; flow: 45 mL×min^-1^, fractionation: 15 mL, gradient: isocratic conditions at 10% B for 10 min, followed by an increase to 70% B in 60 min, then increase from 70% B to 100% B in 5 min, followed by isocratic conditions of 100% B for 5 min. The fractions were combined according to the DAD chromatogram of the HPLC system: flaviolin (**1**): *t*_R_ = 29.5−31.0 min, 18.2 mg; 3,3′-biflaviolin (**2**): *t*_R_ = 38.0−40.0 min, 19.2 mg. Chromatograms, DAD, and MS traces of the pure **1** and **2** are depicted in Figure S2.

Furthermore, other growth media were checked for presence of the isolated compounds **1**−**2**, as well as other compounds of a chemotaxonomic interest. *H.* *invadens* was thus additionally grown in submerged Q6‑½, YM 6.3 (both [45]), MMK2 [46], and HLX [47], as well as the solid medium BRFT [45]. For this purpose, culture conditions as described above were applied, with the difference of only one 500 mL shaking flask being incubated per medium (the solid BRFT medium was also not agitated during cultivation). Extraction of mycelia was conducted as mentioned above, but with only 200 mL of EtOAc being used for ultra-sonication and the acetone ultra-sonication step omitted. Afterwards, the organic EtOAc phase was extracted with ca. 200 mL of water as described above. The supernatant, in turn, was directly extracted with ca. 200 mL of EtOAc each. In the case of BRFT, no separation of supernatant and mycelium was possible due to the solid nature of the medium, hence it was treated like the other mycelia. HPLC‑DAD/MS results of both supernatants and mycelia are depicted in Figure S3.

2.4. Antimicrobial Acticity and Cytotoxicity Assay

Compounds **1**−**2** were dissolved in MeOH (1 mg/mL) for the bioactivity assays. The solvent was also used as negative control.

Minimum inhibitory concentrations (MIC) were determined in a serial dilution assay as described previously [48]. Various test organisms of fungal and bacterial origin were tested to cover a broad range of microorganisms: bacteria: *Bacillus subtilis*, *Staphylococcus aureus*, *Micrococcus luteus*, *Chromobacterium violaceum*, *Escherichia coli, Pseudomonas aeruginosa*; mycobacteria: *Mycolicibacterium smegmatis*; fungi: *Candida albican*s, *Schizosaccharomyces pombe*, *Mucor hiemalis*, *Pichia anomala*, and *Rhodotorula glutinis*.

The MTT cytotoxicity assay was performed against the cell lines L929 (mouse fibroblasts) and KB 3.1 (human papillomavirus-related endocervical adenocarcinoma) as described previously [49].

2.5. Spectral Data

2.5.1. Flaviolin (**1**): Red solid. MW = 206.16 Da, MF = C_10_H_6_O_5_. NMR (acetone-*d*_6_, ^1^H NMR: 500 MHz, ^13^C NMR: 125 MHz): see Table S1; UV/vis (c = 0.01 mg/mL, ACN): *λ*_max_ (*ε*) = 214 (4.41), 262 (3.94), 301 (3.97) nm; ESI−MS: *m/z* 206.92 [M+H]^+^, 204.79 [M−H]^−^; HR−ESI−MS: *m/z* 207.0284 [M+H]^+^ (calculated for C_10_H_7_O_5_, 207.0288); *t*_R_ = 4.8 min.

2.5.2. 3,3′-Biflaviolin (**2**): Red solid. MW = 410.29 Da, MF = C_20_H_10_O_10_. NMR (acetone-*d*_6_, ^1^H NMR: 500 MHz, ^13^C NMR: 125 MHz): see Table S1; UV/vis (c = 0.01 mg/mL, ACN): *λ*_max_ (*ε*) = 211 (4.56), 266 (4.39), 300 (4.15) nm; ESI−MS: *m/z* 411.02 [M+H]^+^, 408.92 [M−H]^−^; HR−ESI−MS: *m/z* 411.0345 [M+H]^+^ (calculated for C_20_H_11_O_10_, 411.0347); *t*_R_ = 6.8 min.

**

**Figure 1.** Inferred Maximum-Likelihood (lLn = −40,100.7533) tree of a molecular phylogeny of sequenced loci of ribosomal origin (ITS, LSU) and proteinogenic genes (RPB2, TUB2), with partition-wise assigned evolutionary models. SH-aLRT (>85%) and BS (>50%) support is given at first and second position at respective branches. The labeled clades correspond to the genera *Annulohypoxylon* (A), *Daldinia* (D), *Entonaema* (E), *Hypoxylon* (Hx 1-7), *Hypomontagnella* (Hy), *Jackrogersella* (J), *Pyrenopolyporus* (P), *Rhopalostroma* (R), *Thamnomyces* (T) and the rooted outgroup (O). The clades have been highlighted by alternative grey and light blue background to make it easier to distinguish them- The position of *H. invadens* is highlighted in **bold**. Note: The branch length of *H. lienhwacheense* has been truncated, as indicated. Branch length equals substitutions per site.

3. Results

3.1. Molecular Phylogeny of H. invadens

The newly generated sequences of *H.* *invadens* and the dataset presented in Table 1 were aligned as described above and curated via Gblocks, which lead to four alignments spanning 347, 1113, 815 and 1047 positions (ITS, LSU, RPB2, and TUB2, respectively; cf. Table S3). In total, 1015 positions were shown as parsimony informative, while 2049 positions represented constant sites. The partitioned analysis (see Table S3 for a list of selected best-fit models following Bayesian-information criterion, BIC) inferred a ML-tree with a log-likelihood (lLn) score of −40,100.7533 (Figure 1). The tree was rooted to the outgroup consisting of representatives of the Graphostromataceae and Xylariaceae (O) with the Hypoxylaceae receiving maximum BS and 99% SH-aLRT support, with *H.* *papillatum* appearing basal to all other clades (Hx1). The Hypoxylaceae furthermore show a paraphyly of *Hypoxylon*, split into one strongly supported clade (Hx2, 98% SH-aLRT / 95% BS) and one strongly supported sister clade (78% BS, Hx7) (85%/56%) put next to *Annulohypoxylon* (A, 100%) and *Jackrogersella* (J, 98%/100%). Furthermore, one non-supported clade containing *H. fragiforme* and allies (Hx3) was placed as sister group next to a clade containing mostly other genera like *Pyrenopolyporus* (P, 100% support), *Daldinia* and allies (99%/100%; E, D, R, T), *Hypomontagnella* (Hy, 100%) and other nested sequences with non- or low supported positions of *Hypoxylon* and *Entonaema* (E, Hx4, Hx5, Hx6) with moderate BS and high likelihood-ratio support (95%/58%). These patterns were already described by Wendt et al. [2], Sir et al. [13], and Pourmoghaddam et al. [23]. The *H.* *invadens*-derived sequences were placed next to *H. trugodes* with maximum support (Hx5).

3.2. Structure Elucidation of Flaviolin (**1**) and 3,3′-Biflaviolin (**2**) and Analysis of Extracts gained from other Culture Media.

**Figure 2.** Chemical structures of flaviolin (**1**) and 3,3′-biflaviolin (**2**), secondary metabolites isolated from cultures of *Hypoxylon invadens.*

Two secondary metabolites were isolated from extracts (*cf.* Figure S1) of cultures of *H. invadens*, which were ultimately identified as the known naphthoquinones flaviolin (**1**) and its dimer 3,3′-biflaviolin (**2**) (see Figure 2). A combination of 1D and 2D NMR as well as a direct comparison of UV/vis spectral data with reference data [50] was applied.

Flaviolin (**1**) was isolated as a red solid and shown to possess a molecular formula (MF) of C_10_H_6_O_5_ by HR‑ESI‑MS. Analysis of 1D and 2D NMR data led to the structure proposal of **1**. The complete structure could be confirmed when different long range coupling constants in ^1^H/^13^C Heteronuclear Multiple-Bond Correlation Spectroscopy (^1^H/^13^C HMBC) experiments (CNST 13 = 2 Hz, 6 Hz) were used, which allowed for ^3^*J* as well as ^4^*J* correlations to be observed. Taken together, these correlations allowed the confirmation of the isolated compound as flaviolin (**1**).

For the red solid 3,3′-biflaviolin (**2**), a MF C_20_H_10_O_10_ was calculated. Its structure was confirmed via NMR spectroscopy to be a symmetrical dimer of **1**. This was deduced by its molecular formula, which represented a duplication of atoms compared to **1** with a formal loss of two hydrogens, highly similar UV/vis spectra (Figure S2), and the similarity of proton and carbon signals in NMR data of **1**−**2**. The bridging atom in 3,3′-biflaviolin was derived to be C‑3/C‑3′, as the ^1^H NMR signals of 3‑H were missing in **2** as compared to **1**.

NMR data, key correlations, as well as 1D and 2D spectra can be found in Table S1, Figure S4 and Figures S5−16, respectively.

The HPLC-DAD/MS chromatograms derived from five screening cultures in six differnet media in which *H. invadens* had been fermented were checked for the presence of compounds **1** and **2**, as well other chemotaxonomically relevant secondary metabolites. Aside from the ZM‑½ medium, where **1** and **2** were major components, we were only able to detect them in HLX medium (*cf.* Figure S3B). Notably, compounds **1** and **2** were not found in extracts from the YM 6.3 medium, which was the standard medium in a comprehensive chemotaxonomic study by Bitzer et al. [19] (Figure S3C). Furthermore, they were neither present in the extracts from submerged MMK2 and Q6‑½ media, nor in the solid, rice-containing BRFT medium (Figure S3D–F). No known compounds from related species (including mellein derivatives, which were present in extracts from submerged cultures in YM medium of almost all *Hypoxylon* sp. previously studied [19], could be assigned to the large number of peaks present in the extracts.

3.3. Antimicrobial and Cytotoxic Activities of Flaviolin (1) and 3,3′-Biflaviolin (2)

The antimicrobial and cytotoxic activities of flaviolin (**1**) and 3,3′-biflaviolin (**2**) were evaluated as described in the Methods Section and are summarized in Table S2.

Antibacterial activity of **1** against *Staphylococcus aureus* with a minimum inhibitory concentration (MIC) of 66.7 µg × mL^−1^ was determined, which was very weak in comparison to the reference oxytetracycline (MIC = 0.4 µg × mL^−1^). No activity of **1**−**2** against any of the other microbial test organisms was observed. Evaluation of the cytotoxic effects of **1**−**2** on mammalian cell lines resulted in **1** exhibiting a half-maximal inhibitory concentration (IC_50_) of 92.2 µM against L929 mouse fibroblasts. The reference epothilone B, in turn, exhibited an IC_50_ of 0.00006 µM against L929. Other cytotoxic effects were not observed against the chosen cell lines.

4. Discussion

In this work, we report the isolation of the first non-volatile secondary metabolites from the fungicolous fungus *Hypoxylon invadens*. To the best of our knowledge, the naphthoquinone polyketides flaviolin (**1**) and its dimer 3,3′-biflaviolin (**2**) have so far neither been described from the genus *Hypoxylon,* nor from any other species of the order Xylariales. During the isolation procedure it became obvious that **1** and **2**, besides some minor components probably representing congeners of those (as inferred from similar DAD and mass spectra), were responsible for the red colour of the culture broth. Even though HPLC‑DAD/MS analysis of the ZM‑½ medium after separation of the mycelium revealed presence of **1** and **2**, quantities were substantially lower than in the mycelium, which was hence used for isolation efforts. Analysis of extracts from other growth media for presence of **1** and **2** revealed that they were also produced in HLX medium. A large number of other peaks were observed in the chromatograms from other cultivation media (Figure S3), but those could not be assigned to known structures solely by analytical methods. Substantial work involving scale up of production and subsequent chromatography and spectral analytics remain necessary to identify these metabolites.

Flaviolin (**1**) has first been described in 1953 by Astill and Roberts [51] as a secondary metabolite from *Aspergillus citricus*, and its structure was elucidated shortly thereafter [52]. In 1979, **1** was deduced to be a melanin biosynthesis shunt product which accumulated when tricyclazole, a fungicide known to inhibit melanin biosynthesis, was added to cultures of *Thielaviopsis basicola* (currently *Berkeleyomyces basicola*) [53]. Biosynthesis of 3,3′-biflaviolin (**2**) was reported to occur when an albino mutant of the fungus was fed with flaviolin (**1**) [50]. In the case of *H.* *invadens* reported herein, **1**−**2** were produced in substantial amounts without intervention in metabolic pathways. This indicates a specific synthesis of **1**−**2** by a polyketide synthase (PKS).

Assessment of the antimicrobial and cytotoxic activity of compounds **1−2** (which was conducted in this study for the first time) showed no significant bioactivities. Only weak antibacterial activities of **1** against *Staphylococcus aureus*, as well as very weak cytotoxicity against the cell line L929 (mouse fibroblasts) was observed. In comparison, no activity against KB 3.1 (human adenocarcinoma) cells was exhibited by **1**, the reason for the differing activities against both eukaryotic cell lines remains unclear. However, the weak bioactivities observed exclude **1**−**2** from further investigation as drug candidates. Extensive assessment of its applicative potential in other assays is currently under way and may reveal more interesting biological effects in the future.

Generally, naphthoquinones are known to be toxic to mammalian cells due to a) their reducibility by biological systems, causing generation of harmful reactive oxygen species (ROS), and b), their ability to covalently bind nucleophiles (such as thiolates of proteins) [54]. While the first effect may readily take place in **1**−**2** due to the 1,4-naphthoquinone moiety, the latter one is unlikely due to the C‑2 hydroxyl moiety in **1** and steric hindrance by the fused ring system in **2** [55]. From an ecological perspective, the role of the herein described secondary metabolites remains obscure for the time being.

Molecular phylogenetic assessment via a multi-locus supermatrix approach led to the placement of *H. invadens* next to *H. trugodes*, (*cf.* Figure 1). However, the exact topological position of the newly formed clade inside the genus *Hypoxylon* lacked statistical support. There are at least 20 species with similar morphology and secondary metabolite profiles as *H. trugodes* (which is a member of the *Hypoxylon rubiginosum* complex) that were not yet subjected to DNA sequencing, and to accomplish this could be an interesting task for the future. With the upcoming genomic era in the study of this diverse genus, *H. invadens* may turn out to become an interesting model species for further ecological, physiological and phylogenomic assessment. Another task for the future would be the biological characterization of the volatile secondary metabolites, which had not been included in the previous study [11]. Notably, these volatiles were identified as terpenoids (e.g., α-muurolene, α-amorphene and α-cadinene), and the chlorinated aromatic compounds, but no naphthoquinone derivatives were found among the volatile metabolites of *H. invadens*. [11].

5. Conclusions

In this work, we report the first description of non-volatile secondary metabolites from the fungicolous *Hypoxylon invadens*. Moreover, these compounds were not found in the over 50 other related species that were studied before, albeit slightly different growth conditions were examined in a previous chemotaxonomic study of related species [19]. However, the large number of unassignable peaks found in extracts of *H.* *invadens* under different conditions suggests isolation efforts of additional secondary metabolites*,* which could, in turn, be used for chemotaxonomic studies and characterized for biological activities. It is remarkable that -despite the fact that several hundreds of secondary metabolites have previously been reported from the Xylariales [6] - the present study is the first to reveal such naphthoquinone derivatives as major components of one of their species.

Moreover, we herein describe the phylogenetic position of *H.* *invadens* in the genus *Hypoxylon*, based on a multi-locus phylogeny. Placement of *H.* *invadens* next to *H.* *trugodes* was well-supported by the applied supermatrix approach (i.e., a new phylogenetic method, which was employed for the Xylariales in this study for the first time), but the exact position of this new clade remains unclear. Further studies, also relying on the newly arising high quality genome sequences that can be generated by third generation sequencing techniques such as Oxford nanopore [7] are called for to further study the ecological role of this interesting fungus and clarify the natural function of its secondary metabolites.

**Supplementary Materials:** The following are available online at www.mdpi.com/xxx/s1, Figure S1: HPLC‑UV/vis Chromatogram of the Crude Extract from Cultures of *H. invadens* in ZM ½ medium used for Isolation of Flaviolin (**1**) and 3,3′-Biflaviolin (**2**); Figure S2: HPLC-UV/vis Chromatograms and DAD/MS Traces of pure **1**−**2**. Figure S3: HPLC-UV/vis Chromatograms of Crude Extracts from Screening Cultures of *H. invadens* in different Media. Table S1: ^1^H and ^13^C NMR data of **1**−**2**; Figure S4: Key NMR correlations of **1**; Table S2: Antimicrobial and cytotoxic activities of **1**−**2**; Figure S5−S10: 1D and 2D NMR spectra of **1**; Figure S11–S16: 1D and 2D NMR spectra of **2**; Table S3: Flanking Positions used for phylogenetic inference as determined by Gblocks and selected evolutionary models. MAFFT alignments of ITS, LSU, RPB2, and TUB2 from *H.* *invadens* are attached as separate FASTA files.

**Author Contributions:** K.B.: resources, investigation, conceptualization, analysis & interpretation of chemical data, visualization, drafting of the paper, critical review of the draft. C.L.: resources, investigation, conceptualization, analysis & interpretation of taxonomical data, data curation, visualization, drafting of the paper, critical review of the draft. J.W.: resources, investigation, analysis & interpretation of data. M.S.: conceptualization, supervision, critical review of the draft.

**Funding:** This research was funded by Deutsche Forschungsgemeinschaft (DFG) in the priority program “Taxon-Omics: New Approaches for Discovering and Naming Biodiversity (SPP 1991)” (K.B. and M.S.). C.L. is thankful for funding by the Life-Science Foundation (Braunschweig, Germany).

**Acknowledgments:** The authors wish to thank Silke Reinecke and Esther Surges for expert assistance in the lab and Christel Kakoschke for recording the NMR spectra. Kirsten Harmrolfs is thanked for support with the structure elucidation.

**Conflicts of Interest:** The authors declare no conflict of interest.

References

1. Hyde, K.D. Refined families of Sordariomycetes. *Mycosphere* **2020**, *11*, 305–1059, doi:10.5943/mycosphere/11/1/7.
2. Wendt, L.; Sir, E.B.; Kuhnert, E.; Heitkämper, S.; Lambert, C.; Hladki, A.I.; Romero, A.I.; Luangsa-ard, J.J.; Srikitikulchai, P.; Peršoh, D., et al. Resurrection and emendation of the Hypoxylaceae, recognised from a multigene phylogeny of the Xylariales. *Mycol. Prog.* **2018**, *17*, 115–154, doi:10.1007/s11557-017-1311-3.
3. Lambert, C.; Wendt, L.; Hladki, A.I.; Stadler, M.; Sir, E.B. *Hypomontagnella* (Hypoxylaceae): a new genus segregated from *Hypoxylon* by a polyphasic taxonomic approach. *Mycol. Prog.* **2018**, *18*, 187–201, doi:10.1007/s11557-018-1452-z.
4. Hsieh, H.M.; Ju, Y.M.; Rogers, J.D. Molecular phylogeny of *Hypoxylon* and closely related genera. *Mycologia* **2005**, *97*, 844–865, doi:10.3852/mycologia.97.4.844.
5. Stadler, M.; Lambert, C.; Wibberg, D.; Kalinowski, J.; Cox, R.J.; Kolařík, M.; Kuhnert, E. Intragenomic polymorphisms in the ITS region of high-quality genomes of the Hypoxylaceae (Xylariales, Ascomycota). *Mycol. Prog.* **2020**, *19*, 235–245, doi:10.1007/s11557-019-01552-9.
6. Helaly, S.E.; Thongbai, B.; Stadler, M. Diversity of biologically active secondary metabolites from endophytic and saprotrophic fungi of the ascomycete order Xylariales. *Nat. Prod. Rep.* **2018**, *35*, 992–1014, doi:10.1039/c8np00010g.
7. Wibberg, D.; Stadler, M.; Lambert, C.; Bunk, B.; Spröer, C.; Rückert, C.; Kalinowski, J.; Cox, R.J.; Kuhnert, E. High quality genome sequences of thirteen Hypoxylaceae (Ascomycota) strengthen the phylogenetic family backbone and enable the discovery of new taxa. *Fungal Divers.* **2020**, doi:10.1007/s13225-020-00447-5.
8. Fournier, J. Two new species of *Hypoxylon* (Xylariaceae) from French Central Pyrénées occurring on *Fagus sylvatica*. *Ascomycete.org* **2014**, *6*, 53–60, doi:10.25664/art-0100.
9. Laessoe, T.; Srikitikulchai, P.; Fournier, J.; Kopcke, B.; Stadler, M. Lepraric acid derivatives as chemotaxonomic markers in *Hypoxylon aeruginosum*, *Chlorostroma subcubisporum* and *C. cyaninum* sp. nov. *Fungal Biol.* **2010**, *114*, 481–489, doi:10.1016/j.funbio.2010.03.010.
10. Dickschat, J.S.; Wang, T.; Stadler, M. Volatiles from the xylarialean fungus *Hypoxylon invadens*. *Beilstein J. Org. Chem.* **2018**, *14*, 734–746, doi:10.3762/bjoc.14.62.
11. Rinkel, J.; Babczyk, A.; Wang, T.; Stadler, M.; Dickschat, J.S. Volatiles from the hypoxylaceous fungi *Hypoxylon griseobrunneum* and *Hypoxylon macrocarpum*. *Beilstein J. Org. Chem.* **2018**, *14*, 2974–2990, doi:10.3762/bjoc.14.277.
12. Kuhnert, E.; Sir, E.B.; Lambert, C.; Hyde, K.D.; Hladki, A.I.; Romero, A.I.; Rohde, M.; Stadler, M. Phylogenetic and chemotaxonomic resolution of the genus *Annulohypoxylon* (Xylariaceae) including four new species. *Fungal Divers.* **2017**, *85*, 1–43, doi:10.1007/s13225-016-0377-6.
13. Sir, E.B.; Becker, K.; Lambert, C.; Bills, G.F.; Kuhnert, E. Observations on Texas hypoxylons, including two new *Hypoxylon* species and widespread environmental isolates of the *H. croceum* complex identified by a polyphasic approach. *Mycologia* **2019**, *111*, 832–856, doi:10.1080/00275514.2019.1637705.
14. Kuhnert, E.; Surup, F.; Halecker, S.; Stadler, M. Minutellins A-D, azaphilones from the stromata of *Annulohypoxylon minutellum* (Xylariaceae). *Phytochemistry* **2017**, *137*, 66–71, doi:10.1016/j.phytochem.2017.02.014.
15. Daranagama, D.A.; Camporesi, E.; Tian, Q.; Liu, X.; Chamyuang, S.; Stadler, M.; Hyde, K.D. *Anthostomella* is polyphyletic comprising several genera in Xylariaceae. *Fungal Divers.* **2015**, *73*, 203–238, doi:10.1007/s13225-015-0329-6.
16. Kuhnert, E.; Heitkamper, S.; Fournier, J.; Surup, F.; Stadler, M. Hypoxyvermelhotins A-C, new pigments from *Hypoxylon lechatii* sp. nov. *Fungal Biol.* **2014**, *118*, 242–252, doi:10.1016/j.funbio.2013.12.003.
17. Triebel, D.; Peršoh, D.; Wollweber, H.; Stadler, M. Phylogenetic relationships among *Daldinia*, *Entonaema*, and *Hypoxylon* as inferred from ITS nrDNA analyses of Xylariales. *Nova Hedw.* **2005**, *80*, 25–43, doi:10.1127/0029-5035/2005/0080-0025.
18. Stadler, M.; Laessoe, T.; Fournier, J.; Decock, C.; Schmieschek, B.; Tichy, H.V.; Persoh, D. A polyphasic taxonomy of *Daldinia* (Xylariaceae). *Stud. Mycol.* **2014**, *77*, 1–143, doi:10.3114/sim0016.
19. Bitzer, J.; Laessoe, T.; Fournier, J.; Kummer, V.; Decock, C.; Tichy, H.V.; Piepenbring, M.; Persoh, D.; Stadler, M. Affinities of *Phylacia* and the daldinoid Xylariaceae, inferred from chemotypes of cultures and ribosomal DNA sequences. *Mycol. Res.* **2008**, *112*, 251–270, doi:10.1016/j.mycres.2007.07.004.
20. Zhang, N.; Castlebury, L.A.; Miller, A.N.; Huhndorf, S.M.; Schoch, C.L.; Seifert, K.A.; Rossman, A.Y.; Rogers, J.D.; Kohlmeyer, J.; Volkmann-Kohlmeyer, B., et al. An overview of the systematics of the Sordariomycetes based on a four-gene phylogeny. *Mycologia* **2017**, *98*, 1076–1087, doi:10.1080/15572536.2006.11832635.
21. Koukol, O.; Kelnarová, I.; Černý, K.; Woodward, S. Recent observations of sooty bark disease of sycamore maple in Prague (Czech Republic) and the phylogenetic placement of *Cryptostroma corticale*. *For. Pathol.* **2015**, *45*, 21–27, doi:10.1111/efp.12129.
22. Stadler, M.K., Eric ; Peršoh, Derek ; Fournier, Jacques. The Xylariaceae as model example for a unified nomenclature following the “One Fungus-One Name” (1F1N) concept. *Mycology* **2013**, *4*, 5–21, doi:10.1080/21501203.2013.782478.
23. Pourmoghaddam, M.J.; Lambert, C.; Surup, F.; Khodaparast, S.A.; Krisai-Greilhuber, I.; Voglmayr, H.; Stadler, M. Discovery of a new species of the *Hypoxylon rubiginosum* complex from Iran and antagonistic activities of *Hypoxylon* spp. against the Ash Dieback pathogen, *Hymenoscyphus fraxineus*, in dual culture. *MycoKeys* **2020**, *66*, 105–133, doi:10.3897/mycokeys.66.50946.
24. Li, G.J.; Hyde, K.D.; Zhao, R.L.; Hongsanan, S.; Abdel-Aziz, F.A.; Abdel-Wahab, M.A.; Alvarado, P.; Alves-Silva, G.; Ammirati, J.F.; Ariyawansa, H.A., et al. Fungal diversity notes 253–366: taxonomic and phylogenetic contributions to fungal taxa. *Fungal Divers.* **2016**, *78*, 1–237, doi:10.1007/s13225-016-0366-9.
25. Kuhnert, E.; Fournier, J.; Peršoh, D.; Luangsa-ard, J.J.D.; Stadler, M. New *Hypoxylon* species from Martinique and new evidence on the molecular phylogeny of *Hypoxylon* based on ITS rDNA and *β*-tubulin data. *Fungal Divers.* **2013**, *64*, 181–203, doi:10.1007/s13225-013-0264-3.
26. Bills, G.F.; Gonzalez-Menendez, V.; Martin, J.; Platas, G.; Fournier, J.; Persoh, D.; Stadler, M. *Hypoxylon pulicicidum* sp. nov. (Ascomycota, Xylariales), a pantropical insecticide-producing endophyte. *PLoS One* **2012**, *7*, e46687, doi:10.1371/journal.pone.0046687.
27. Stadler, M.; Flessa, F.; Rambold, G.; Peršoh, D.; Stadler, M.; Fournier, J.; Læssøe, T.; Chlebicki, A.; Lechat, C.; Peršoh, D. Chemotaxonomic and phylogenetic studies of *Thamnomyces* (Xylariaceae). *Mycoscience* **2010**, *51*, 189–207, doi:10.1007/s10267-009-0028-9.
28. Sir, E.B. A new species of *Daldinia* (Xylariaceae) from the Argentine subtropical montane forest. *Mycosphere* **2016**, *7*, 1378–1388, doi:10.5943/mycosphere/7/9/11.
29. White, T.J.; Bruns, T.; Lee, S.; Taylor, J. Amplification and direct sequencing of fungal ribosomal RNA genes for phylogenetics. In *PCR Protocols*, Academic Press: Cambridge, MA, USA, 1990; pp. 315-322.
30. Gardes, M.; Bruns, T.D. ITS primers with enhanced specificity for basidiomycetes-application to the identification of mycorrhizae and rusts. *Mol. Ecol.* **1993**, *2*, 113–118, doi:10.1111/j.1365-294x.1993.tb00005.x.
31. Vilgalys, R.; Hester, M. Rapid genetic identification and mapping of enzymatically amplified ribosomal DNA from several *Cryptococcus* species. *J. Bacteriol.* **1990**, *172*, 4238–4246, doi:10.1128/jb.172.8.4238-4246.1990.
32. Liu, Y.J.; Whelen, S.; Hall, B.D. Phylogenetic relationships among ascomycetes: evidence from an RNA polymerse II subunit. *Mol. Biol. Evol.* **1999**, *16*, 1799–1808, doi:10.1093/oxfordjournals.molbev.a026092.
33. O’Donnell, K.; Cigelnik, E. Two divergent intragenomic rDNA ITS2 types within a monophyletic lineage of the fungus *Fusarium* are nonorthologous. *Mol. Phylogenet. Evol.* **1997**, *7*, 103–116, doi:10.1006/mpev.1996.0376.
34. Otto, A.; Laub, A.; Wendt, L.; Porzel, A.; Schmidt, J.; Palfner, G.; Becerra, J.; Kruger, D.; Stadler, M.; Wessjohann, L., et al. Chilenopeptins A and B, peptaibols from the Chilean *Sepedonium aff. chalcipori* KSH 883. *J. Nat. Prod.* **2016**, *79*, 929–938, doi:10.1021/acs.jnatprod.5b01018.
35. Kearse, M.; Moir, R.; Wilson, A.; Stones-Havas, S.; Cheung, M.; Sturrock, S.; Buxton, S.; Cooper, A.; Markowitz, S.; Duran, C., et al. Geneious Basic: an integrated and extendable desktop software platform for the organization and analysis of sequence data. *Bioinformatics* **2012**, *28*, 1647–1649, doi:10.1093/bioinformatics/bts199.
36. Katoh, K.; Misawa, K.; Kuma, K.; Miyata, T. MAFFT: a novel method for rapid multiple sequence alignment based on fast Fourier transform. *Nucleic Acids Res.* **2002**, *30*, 3059–3066, doi:10.1093/nar/gkf436.
37. Castresana, J. Selection of conserved blocks from multiple alignments for their use in phylogenetic analysis. *Mol. Biol. Evol.* **2000**, *17*, 540–552, doi:10.1093/oxfordjournals.molbev.a026334.
38. Talavera, G.; Castresana, J. Improvement of phylogenies after removing divergent and ambiguously aligned blocks from protein sequence alignments. *Syst. Biol.* **2007**, *56*, 564–577, doi:10.1080/10635150701472164.
39. Minh, B.Q.; Schmidt, H.A.; Chernomor, O.; Schrempf, D.; Woodhams, M.D.; von Haeseler, A.; Lanfear, R. IQ-TREE 2: new models and efficient methods for phylogenetic inference in the genomic era. *Mol. Biol. Evol.* **2020**, *37*, 1530–1534, doi:10.1093/molbev/msaa015.
40. Chernomor, O.; von Haeseler, A.; Minh, B.Q. Terrace aware data structure for phylogenomic inference from supermatrices. *Syst. Biol.* **2016**, *65*, 997–1008, doi:10.1093/sysbio/syw037.
41. Kalyaanamoorthy, S.; Minh, B.Q.; Wong, T.K.F.; von Haeseler, A.; Jermiin, L.S. ModelFinder: fast model selection for accurate phylogenetic estimates. *Nat. Methods* **2017**, *14*, 587–589, doi:10.1038/nmeth.4285.
42. Felsenstein, J. Confidence limits on phylogenies: an approach using the bootstrap. *Evolution* **1985**, *39*, 783–791, doi:10.1111/j.1558-5646.1985.tb00420.x.
43. Guindon, S.; Dufayard, J.F.; Lefort, V.; Anisimova, M.; Hordijk, W.; Gascuel, O. New algorithms and methods to estimate maximum-likelihood phylogenies: assessing the performance of PhyML 3.0. *Syst. Biol.* **2010**, *59*, 307–321, doi:10.1093/sysbio/syq010.
44. Shimodaira, H.; Hasegawa, M. Multiple comparisons of log-likelihoods with applications to phylogenetic inference. *Mol. Biol. Evol.* **1999**, *16*, 1114–1116, doi:10.1093/oxfordjournals.molbev.a026201.
45. Becker, K.; Wongkanoun, S.; Wessel, A.-C.; Bills, G.F.; Stadler, M.; Luangsa-ard, J.J. Phylogenetic and chemotaxonomic studies confirm the affinities of *Stromatoneurospora phoenix* to the coprophilous Xylariaceae. *J. Fungi* **2020**, *6*, doi:10.3390/jof6030144.
46. Gonzalez-Menendez, V.; Asensio, F.; Moreno, C.; de Pedro, N.; Monteiro, M.C.; de la Cruz, M.; Vicente, F.; Bills, G.F.; Reyes, F.; Genilloud, O., et al. Assessing the effects of adsorptive polymeric resin additions on fungal secondary metabolite chemical diversity. *Mycology* **2014**, *5*, 179–191, doi:10.1080/21501203.2014.942406.
47. Stadler, M.; Tichy, H.-V.; Katsiou, E.; Hellwig, V. Chemotaxonomy of *Pochonia* and other conidial fungi with *Verticillium*-like anamorphs. *Mycol. Prog.* **2003**, *2*, 95–122, doi:10.1007/s11557-006-0048-1.
48. Becker, K.; Wessel, A.-C.; Luangsa-ard, J.J.; Stadler, M. Viridistratins A−C, antimicrobial and cytotoxic benzo[*j*]fluoranthenes from stromata of *Annulohypoxylon viridistratum* (Hypoxylaceae, Ascomycota) *Biomolecules* **2020**, *10*, doi:doi.org/10.3390/biom10050805.
49. Sandargo, B.; Michehl, M.; Praditya, D.; Steinmann, E.; Stadler, M.; Surup, F. Antiviral meroterpenoid rhodatin and sesquiterpenoids rhodocoranes A-E from the Wrinkled Peach Mushroom, *Rhodotus palmatus*. *Org. Lett.* **2019**, *21*, 3286–3289, doi:10.1021/acs.orglett.9b01017.
50. Stipanovic, R.D.; Wheeler, M.H. Accumulation of 3,3′-biflaviolin, a melanin shunt product, by tricyclazole-treated *Thielaviopsis basicola*. *Pestic. Biochem. Physiol.* **1980**, *13*, 198-201, doi:10.1016/0048-3575(80)90072-3.
51. Astill, B.D.; Roberts, J.C. 654. Studies in mycological chemistry. Part I. Flaviolin, 2(or 3):5:7-trihydroxy-1:4-naphthaquinone, a metabolic product of *Aspergillus citricus* (Wehmer) Mosseray. *J. Chem. Soc.* **1953**, doi:10.1039/jr9530003302.
52. Davies, J.E.; King, F.E.; Roberts, J.C. The structure of flaviolin. *Chem. Ind.* **1954**, 1110–1111, doi.
53. Wheeler, M.H.; Stipanovic, R.D. Melanin biosynthesis in *Thielaviopsis basicola*. *Exp. Mycol.* **1979**, *3*, 340-350, doi:10.1016/s0147-5975(79)80037-7.
54. Kumagai, Y.; Shinkai, Y.; Miura, T.; Cho, A.K. The chemical biology of naphthoquinones and its environmental implications. *Annu. Rev. Pharmacol. Toxicol.* **2012**, *52*, 221–247, doi:10.1146/annurev-pharmtox-010611-134517.
55. Klotz, L.O.; Hou, X.; Jacob, C. 1,4-naphthoquinones: from oxidative damage to cellular and inter-cellular signaling. *Molecules* **2014**, *19*, 14902–14918, doi:10.3390/molecules190914902.

| 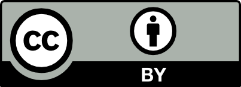 | © 2020 by the authors. Submitted for possible open access publication under the terms and conditions of the Creative Commons Attribution (CC BY) license (http://creativecommons.org/licenses/by/4.0/). |
| --- | --- |
