# Supplementary material for: Phylogenetic Assignment of the Fungicolous Hypoxylon invadens (Ascomycota, Xylariales) and Investigation of its Secondary Metabolites
Source: Microorganisms. 2020 Sep 11;8(9):1397. doi: 10.3390/microorganisms8091397 (PMC7565716; doi:10.3390/microorganisms8091397)
Supplement: Supplementary file 1 [file microorganisms-08-01397-s001.zip › microorganisms-892779 supple and non published/microorganisms-892779 proofed supplementary.pdf]

Supporting Information

Phylogenetic Assignment of the fungicolous *Hypoxylon invadens* (Ascomycota, Xylariales) and Investigation into its Secondary Metabolism

Kevin Becker [1,2], Christopher Lambert [1,2,3], Jörg Wieschhaus [1], Marc Stadler [1,2,*]

[1] Department of Microbial Drugs, Helmholtz Centre for Infection Research GmbH (HZI), Inhoffenstraße 7, 38124 Braunschweig, Germany; kevin.becker@helmholtz-hzi.de (K.B.), christopher.lambert@helmholtz-hzi.de (C.L.), joerg.wieschhaus@gmail.com (J.W.)

[2] German Centre for Infection Research Association (DZIF), Partner site Hannover-Braunschweig, Inhoffenstraße 7, 38124 Braunschweig, Germany

[3] Department for Molecular Cell Biology, Helmholtz Centre for Infection Research GmbH (HZI) Inhoffenstraße 7, 38124 Braunschweig, Germany

* Correspondence: marc.stadler@helmholtz-hzi.de (M.S.); Tel.: +49-531-6181-4240; Fax: +49-531-6181-9499

Contents

Figure S1: HPLC-UV/vis Chromatogram of the Crude Extract from Cultures of *H. invadens* in ZM-½ medium used for Isolation of Flaviolin (**1**) and 3,3′-Biflaviolin (**2**) 2

Figure S2: HPLC-UV/vis Chromatograms and DAD/MS Traces of pure **1**–**2** 2

Figure S3: HPLC-UV/vis Chromatograms of Crude Extracts from Screening Cultures of *H. invadens* in different Media 3

Table S1: NMR Chemical Shifts of **1**–**2** 5

Figure S4: Key NMR Correlations of **1** 5

Table S2: Antimicrobial and Cytotoxic Activities of **1**–**2** 6

Figures S5–S10: 1D and 2D NMR Spectra of **1** 7

Figures S11–S16: 1D and 2D NMR Spectra of **2** 13

Table S3: Flanking Positions used for phylogenetic inference as determined by Gblocks and selected evolutionary models 19

MAFFT Alignments of ITS, LSU, RPB2, TUB2 gene regions 19

Figure S1: HPLC-UV/vis Chromatogram of the Crude Extract from Cultures of *H. invadens* in ZM-½ medium used for Isolation of Flaviolin (1) and 3,3′-Biflaviolin (2)

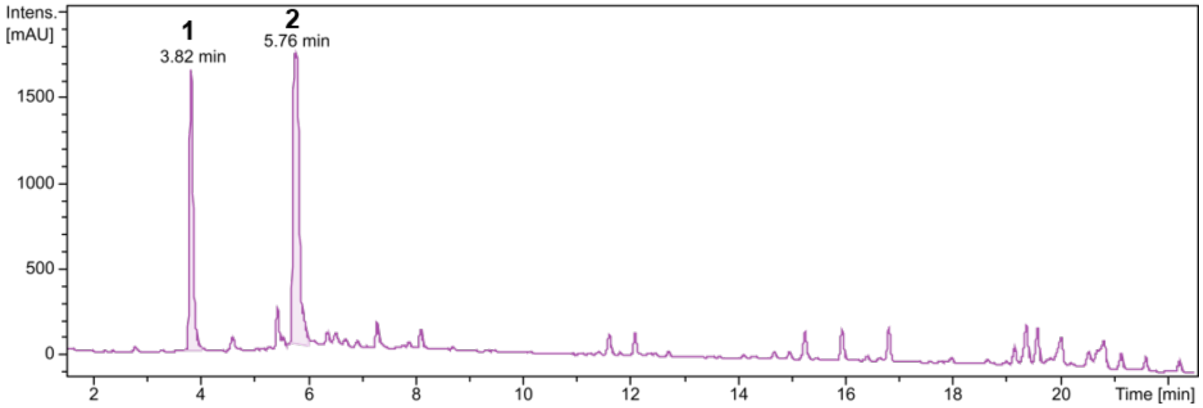

Figure S1: HPLC-UV/vis chromatogram at 210 nm of the combined crude extract from subsequent extractions with EtOAc and acetone, gained from cultures of *Hypoxylon invadens* in ZM-½ medium. Labels indicate flaviolin (**1**) and 3,3′-biflaviolin (**2**). Note: retention times depicted here differ from those in Figure S2 due to usage of different HPLC systems.

Figure S2: HPLC-UV/vis Chromatograms and DAD/MS Traces of pure 1–2

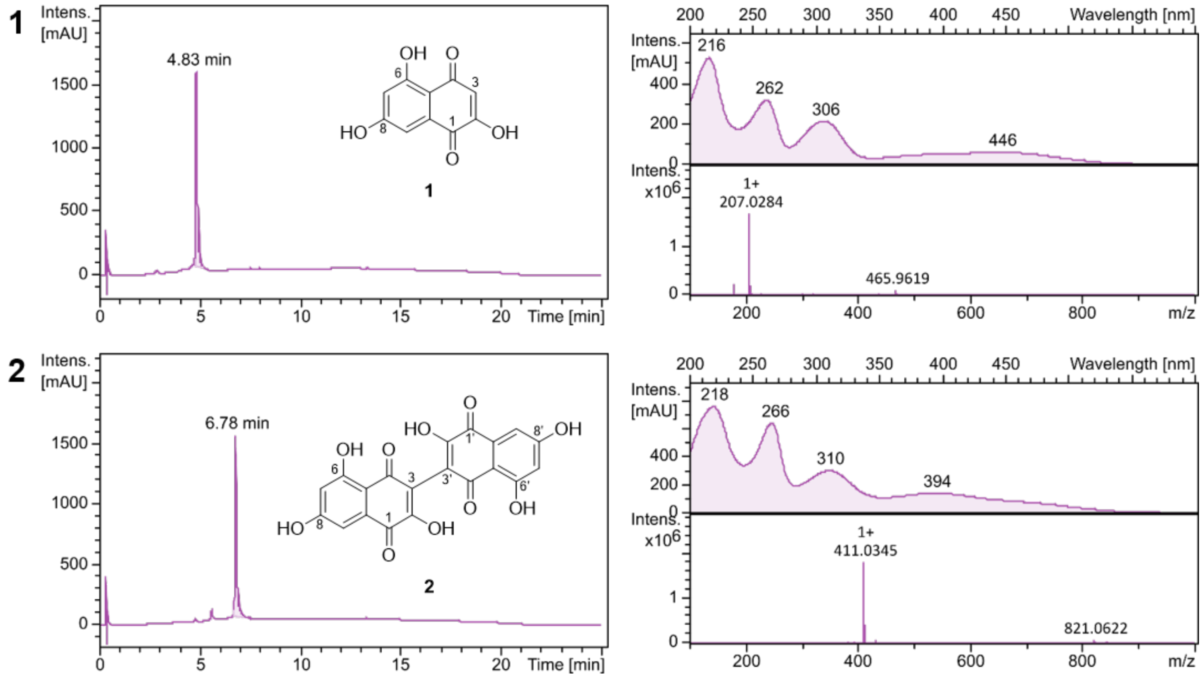

Figure S2: HPLC-UV/vis chromatograms at 210 nm, DAD and HR-ESI-MS(+) traces of flaviolin (**1**) and 3,3′-biflaviolin (**2**). Note: retention times depicted here differ from those in Figure S1 and Figure S3 due to usage of different HPLC systems.

Figure S3: HPLC-UV/vis Chromatograms of Crude Extracts from Screening Cultures of *H. invadens* in different Media

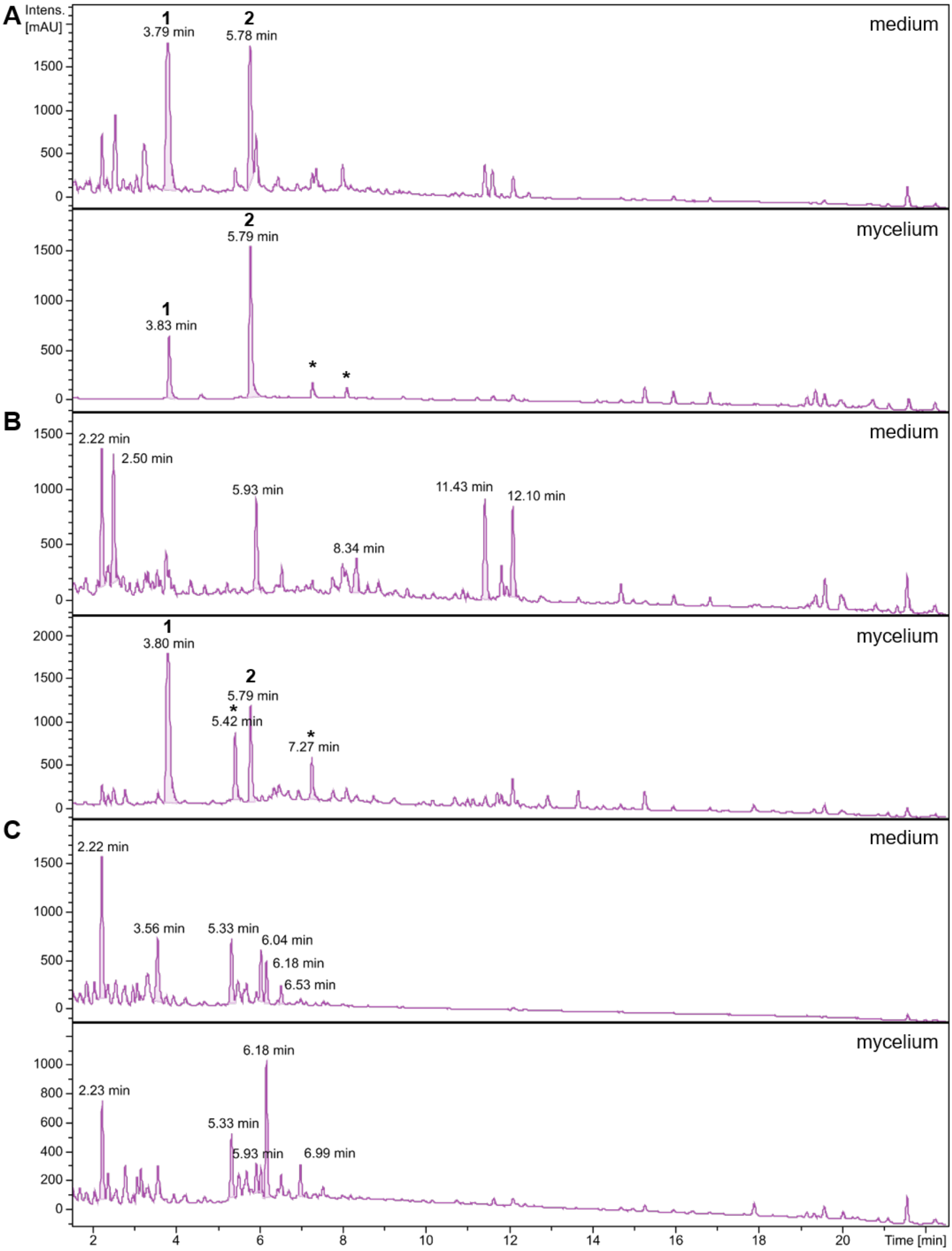

Figure S3: HPLC-UV/vis chromatograms at 210 nm of crude extracts gained from screening cultures of *Hypoxylon invadens* in different growth media. **A**: ZM-½; **B**: HLX; **C**: YM 6.3. Note: retention times depicted here differ from those in Figure S2 due to usage of different HPLC systems. Labels indicate flaviolin (**1**) and 3,3′-biflaviolin (**2**), as well as presumed congeners of **1**–**2** (*) derived from MS and UV/vis data.

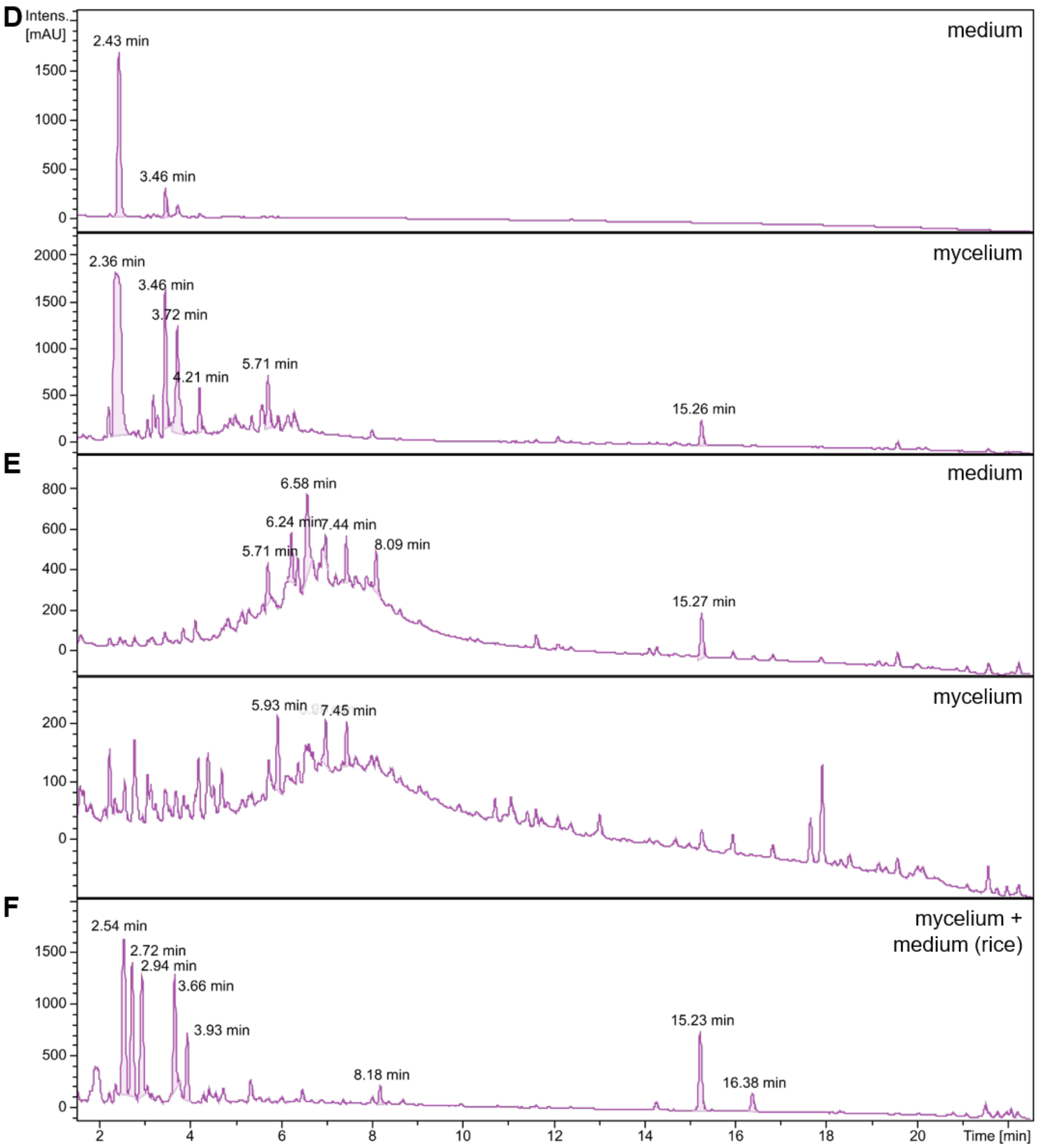

Figure S3 (continued): HPLC-UV/vis chromatograms at 210 nm of crude extracts gained from screening cultures of *Hypoxylon invadens* in different growth media. **D**: MMK2; **E**: Q6-½; **F**: BRFT (solid medium). Note: retention times depicted here differ from those in Figure S2 due to usage of different HPLC systems.

Table S1: NMR Chemical Shifts of 1–2

Table S1. ^{1}H and ^{13}C NMR data of flaviolin (**1**) and 3,3′-biflaviolin (**2**) (acetone-d_6, ^{1}H NMR: 500 MHz, ^{13}C NMR: 125 MHz)

**1**			**2**		
**pos** [1]	**δ_C, mult** [2]	**δ_H, mult** [2]	**pos**	**δ_C, mult**	**δ_H, mult**
1	181.8, C		1/1′	181.2, C	
2	159.8, C		2/2′	157.4, C	
3	111.2, CH	6.11, s	3/3′	115.0, C	
4	191.4, C		4/4′	188.7, C	
5	109.1, C		5/5′	109.1, C	
6	164.7, C		6/6′	165.0, C	
7	109.6, CH	6.61, d (2.29)	7/7′	109.8, CH	6.67, d (2.29)
8	164.5, C		8/8′	164.7, C	
9	109.0, CH	7.08, d (2.29)	9/9′	109.2, CH	7.17, d (2.29)
10	133.2, C		10/10′	133.1, C	
6-OH	-	12.53, s	6/6′-OH	-	12.52, s

[1] pos: atom position (see Figure S2); [2] δ_C/δ_H: chemical shift [ppm]; mult: multiplicity, s: singlet, d: doublet.

Figure S4: Key NMR Correlations of 1

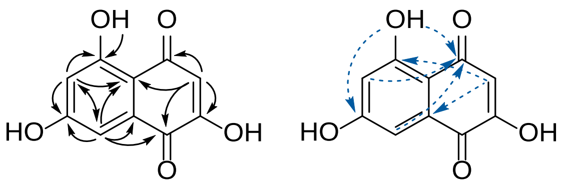

Figure S4. Key NMR correlations of flaviolin (**1**). Plain arrows: ^{2}J and ^{3}J ^{1}H/^{13}C HMBC correlations (left); dashed, blue arrows: ^{4}J ^{1}H/^{13}C HMBC correlations (right).

Table S2: Antimicrobial and Cytotoxic Activities of 1–2

Table S2. Antimicrobial and cytotoxic activities of flaviolin (**1**) and 3,3′-biflaviolin (**2**). Top panel: antimicrobial activities as minimum inhibitory concentrations (MIC); bottom: cytotoxicities as half-maximal inhibitory concentrations (IC_{50}). n.i.: no inhibition observed.

test organism / cell line	MIC [µg/mL] / IC_{50} [µM]		
	1	2	reference
Bacillus subtilis	>66.7	>66.7	8.3 [1]
Staphylococcus aureus	66.7	>66.7	0.4 [2]
Micrococcus luteus	>66.7	>66.7	0.8 [2]
Chromobacterium violaceum	>66.7	>66.7	0.1 [2]
Escherichia coli	>66.7	>66.7	1.7 [2]
Pseudomonas aeruginosa	>66.7	>66.7	0.4 [3]
Mycolicibacterium smegmatis	>66.7	>66.7	3.3 [4]
Candida albicans	>66.7	>66.7	66.7 [5]
Schizosaccharomyces pombe	>66.7	>66.7	33.3 [5]
Mucor hiemalis	>66.7	>66.7	66.7 [5]
Pichia anomala	>66.7	>66.7	66.7 [5]
Rhodotorula glutinis	>66.7	>66.7	16.7 [5]
L929 (mouse fibroblasts)	92.2	n.i.	0.00006 [6]
KB 3.1 (human endocervical adenocarcinoma)	n.i.	n.i.	0.00079 [6]

[1] oxytetracycline: 1 mg/mL, [2] oxytetracycline: 0.1 mg/mL, [3] gentamicin: 0.1 mg/mL, [4] kanamycin: 0.1 mg/mL, [5] nystatin: 1 mg/mL, [6] epothilone B: 1 mg/mL.

Figures S5–S10: 1D and 2D NMR Spectra of 1

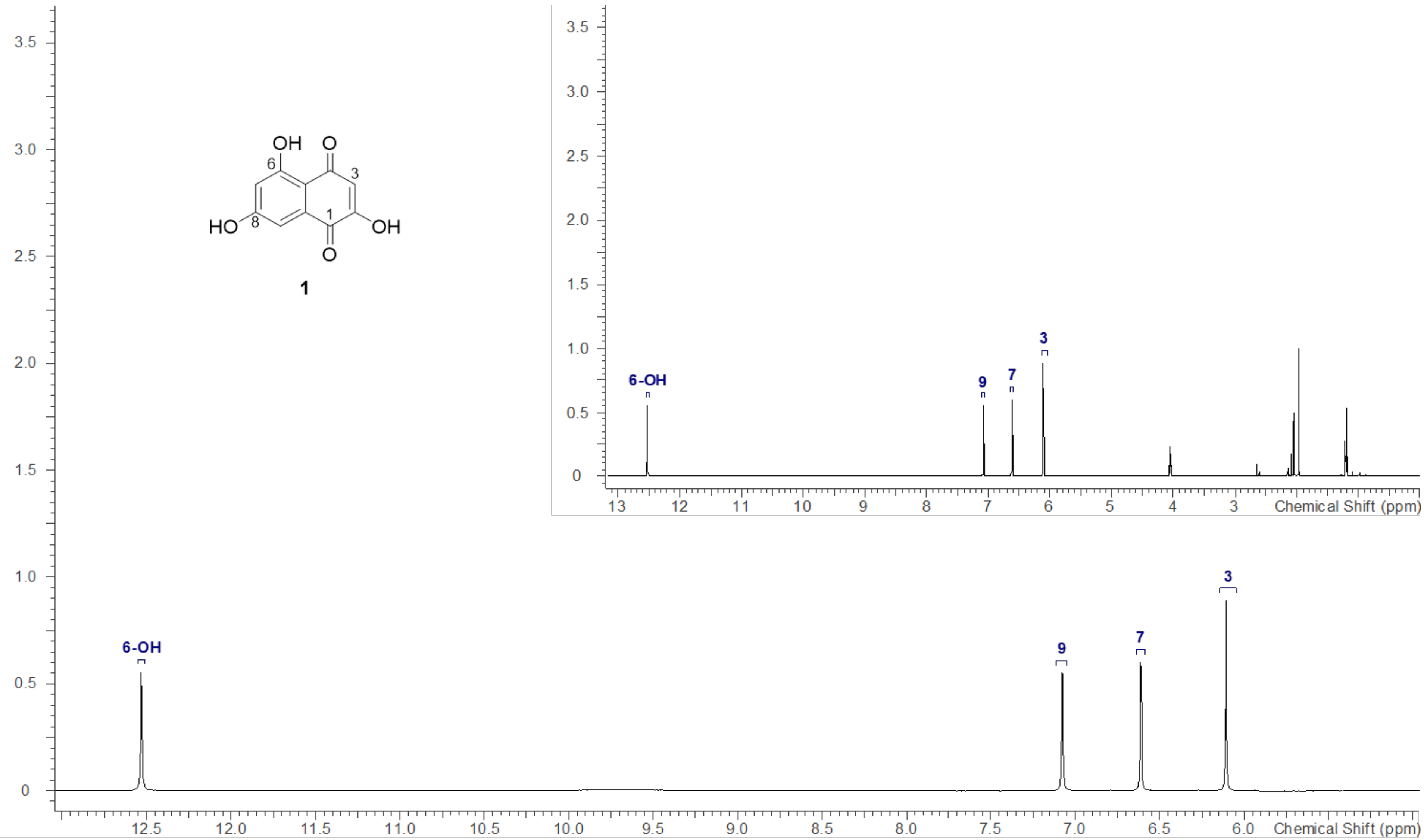

Figure S5: ^{1}H NMR spectrum (500 MHz, acetone-d_6) of flaviolin (**1**). Inner panel: complete spectrum view.

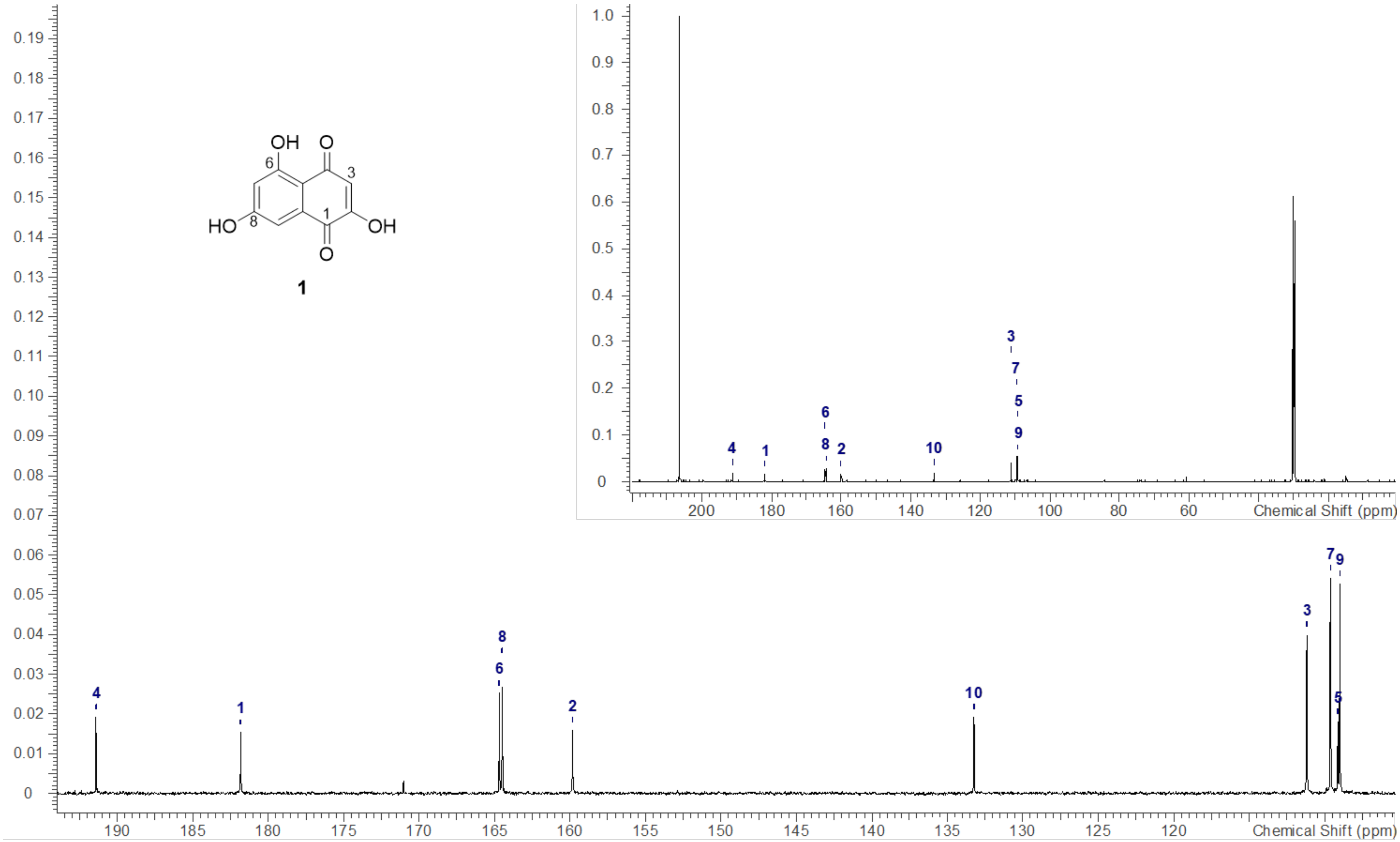

Figure S6: ^{13}C NMR spectrum (125 MHz, acetone-d_6) of flaviolin (**1**). Inner panel: complete spectrum view

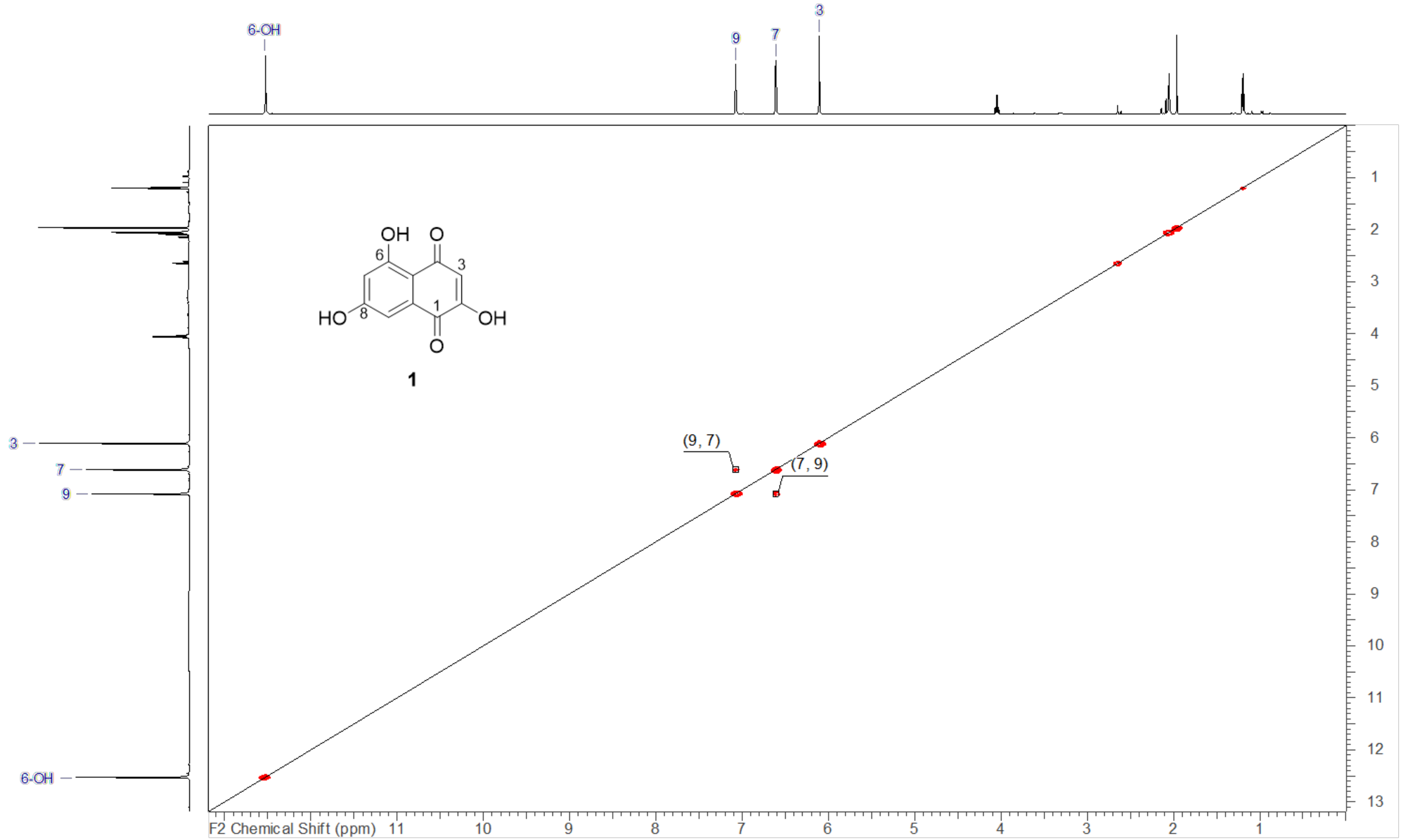

Figure S7: $^1H/^1H$ COSY spectrum (500 MHz, acetone-*d*$_6$) of flaviolin (**1**).

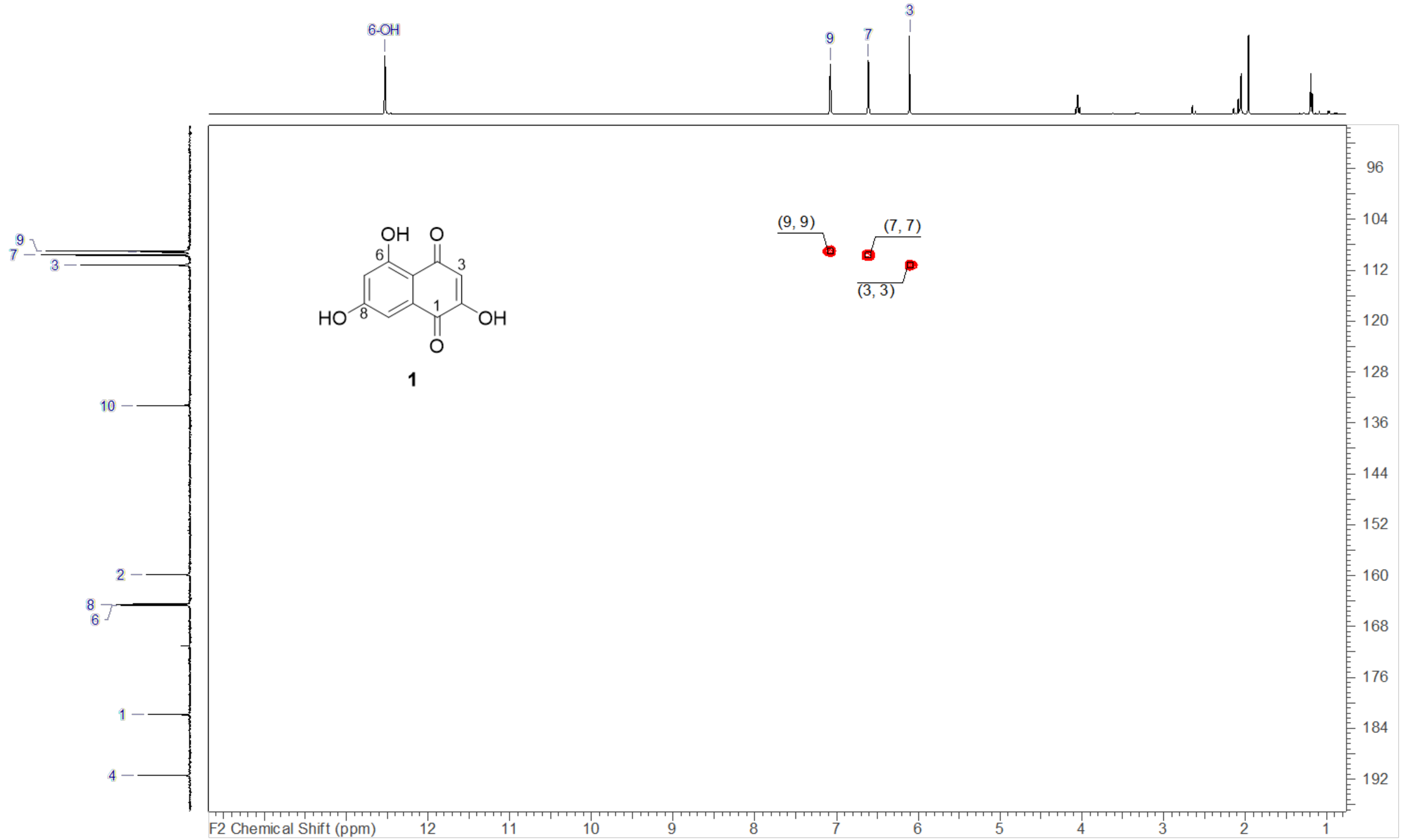

Figure S8: $^{1}H/^{13}C$ HSQC spectrum (500 MHz, acetone-d_6) of flaviolin (**1**).

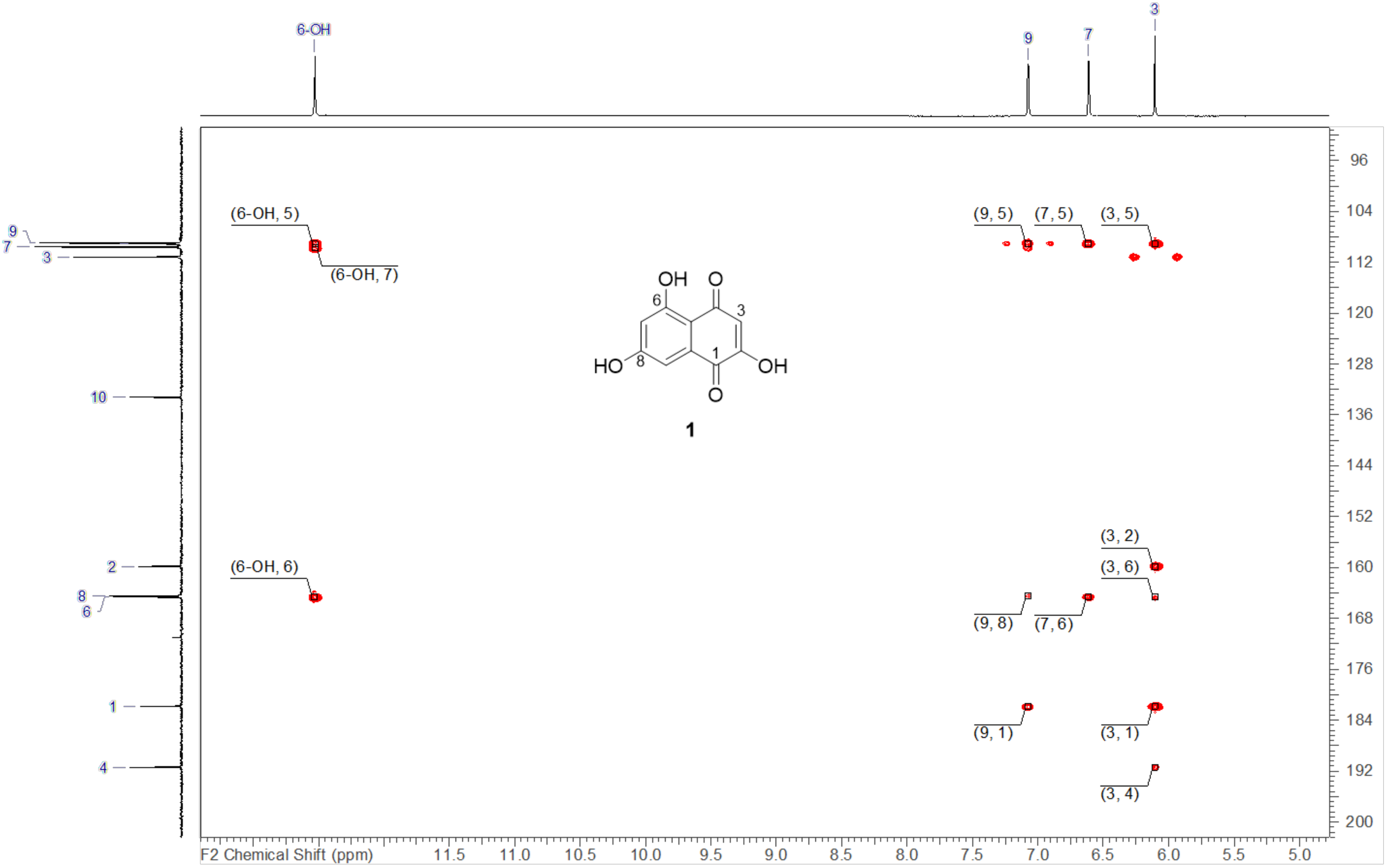

Figure S9: $^{1}H/^{13}C$ HMBC spectrum (500 MHz, acetone-d_6) of flaviolin (**1**) (CNST 13 = 6 Hz).

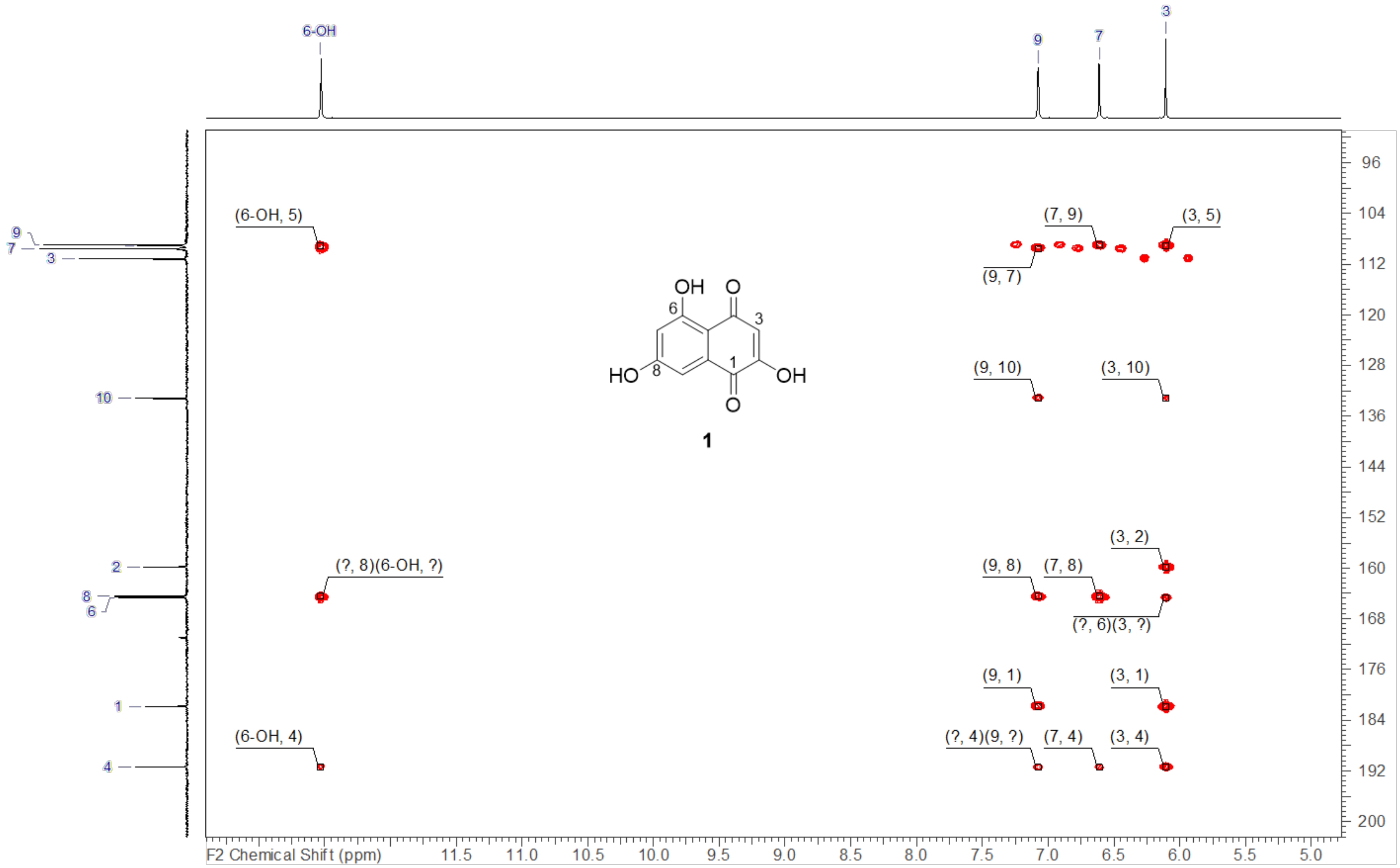

Figure S10: $^1H/^{13}C$ HMBC spectrum (500 MHz, acetone-d_6) of flaviolin (**1**) (CNST 13 = 2 Hz).

Figures S11–S16: 1D and 2D NMR Spectra of 2

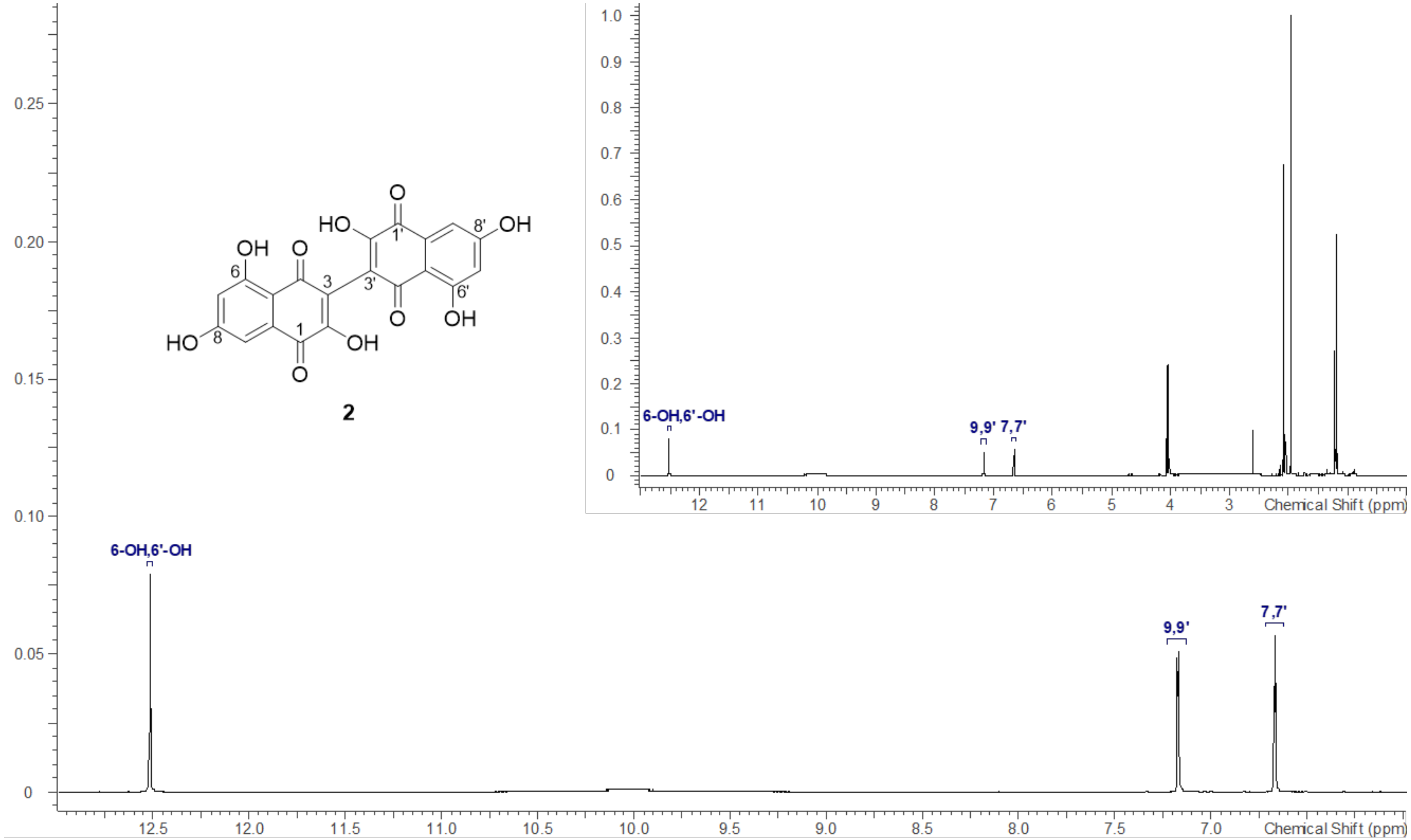

Figure S11: ^{1}H NMR spectrum (500 MHz, acetone-d_6) of 3,3′-biflaviolin (**2**).

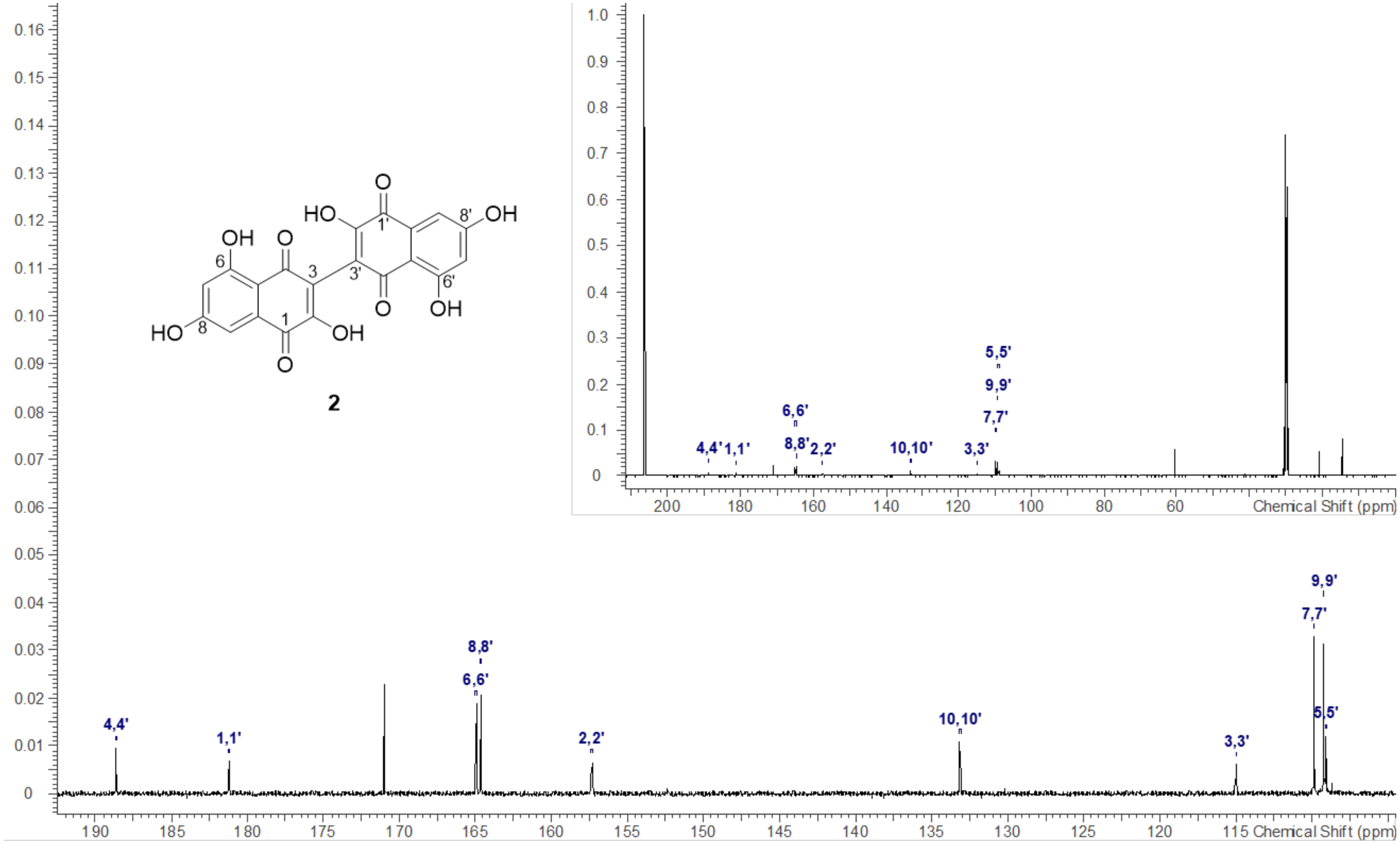

Figure S12: ^{13}C NMR spectrum (125 MHz, acetone-d_6) of 3,3′-biflaviolin (**2**).

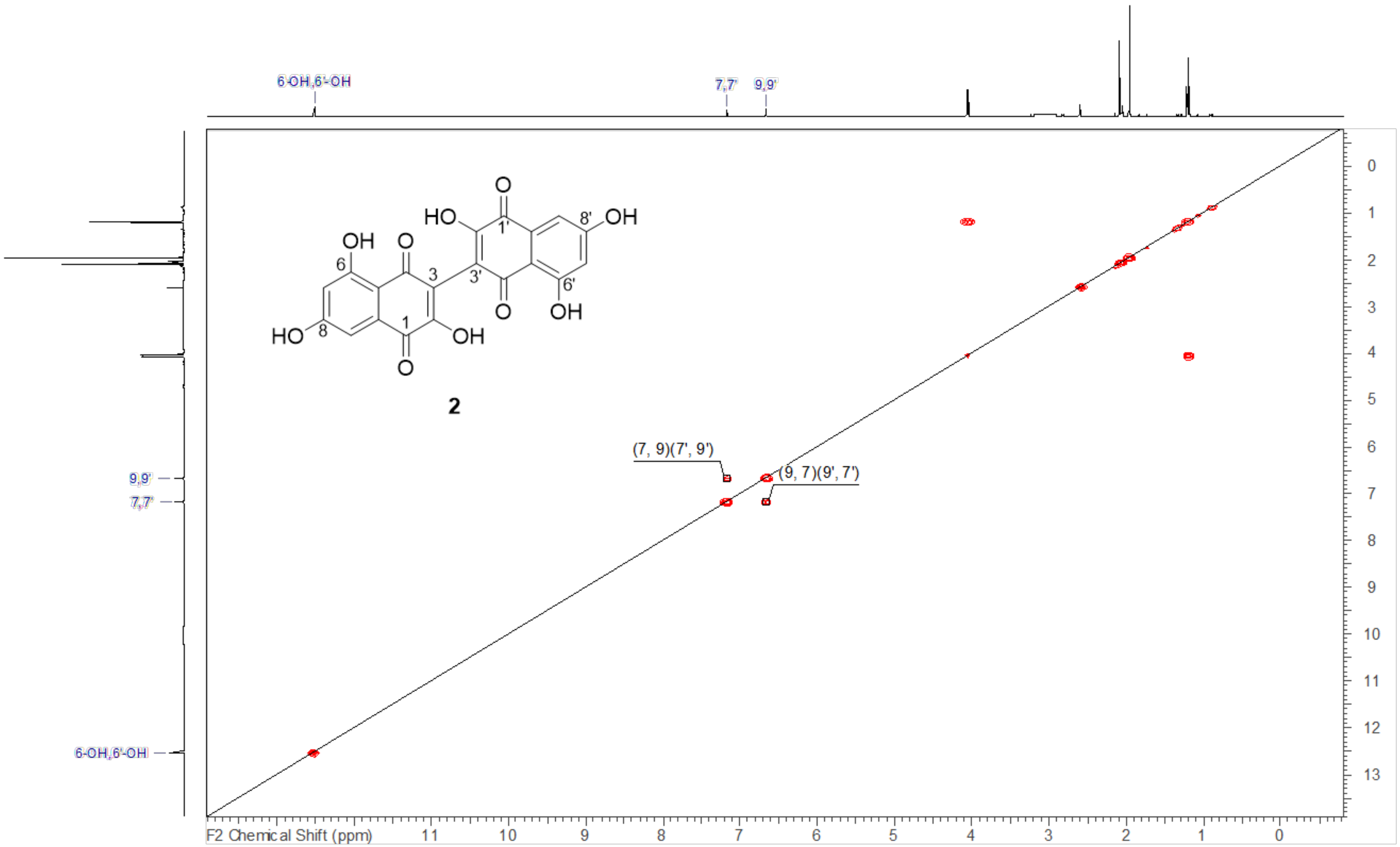

Figure S13: $^1H/^1H$ COSY spectrum (500 MHz, acetone-d_6) of 3,3′-biflaviolin (**2**).

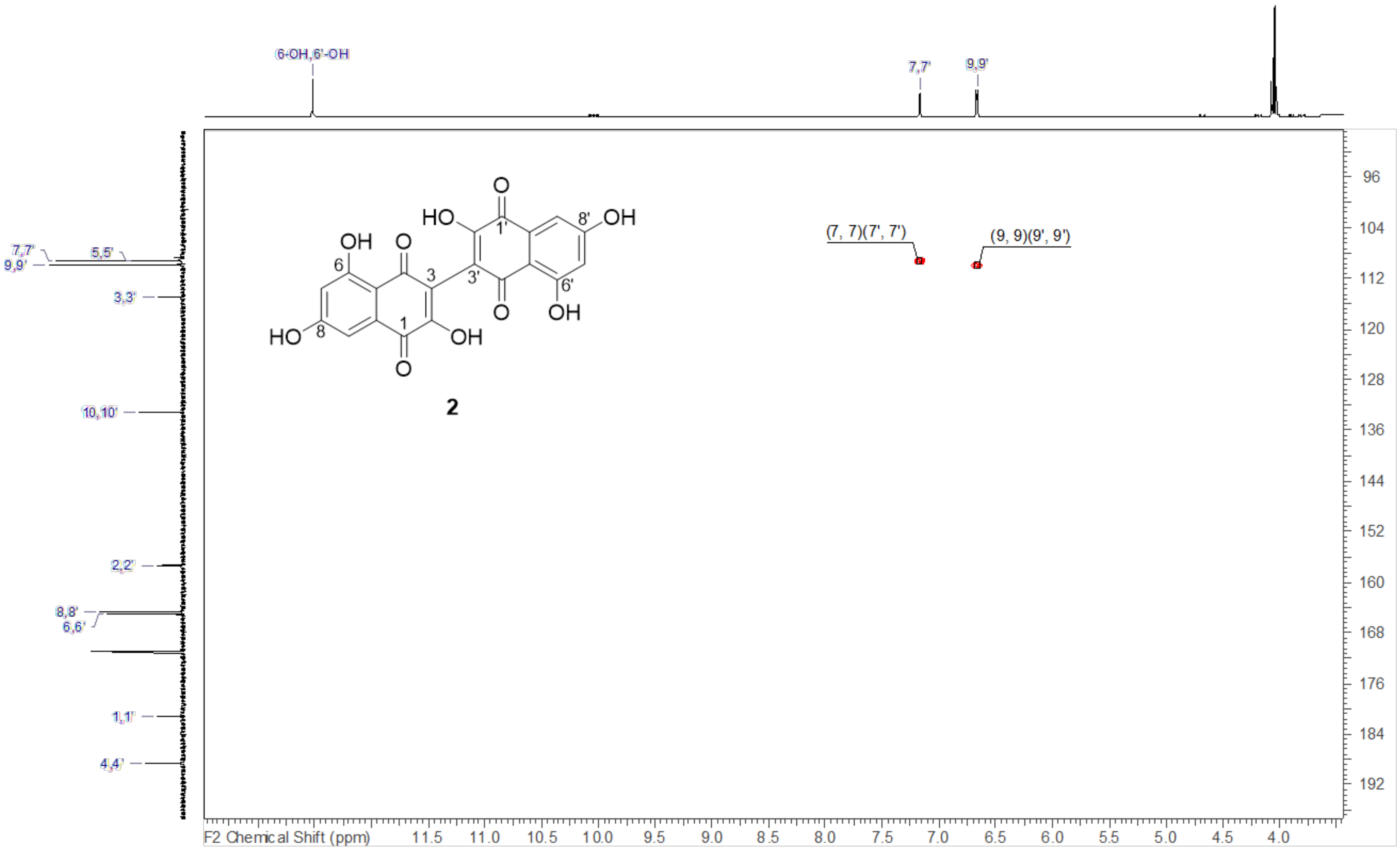

Figure S14: $^1H/^{13}C$ HSQC spectrum (500 MHz, acetone-d_6) of 3,3′-biflaviolin (**2**).

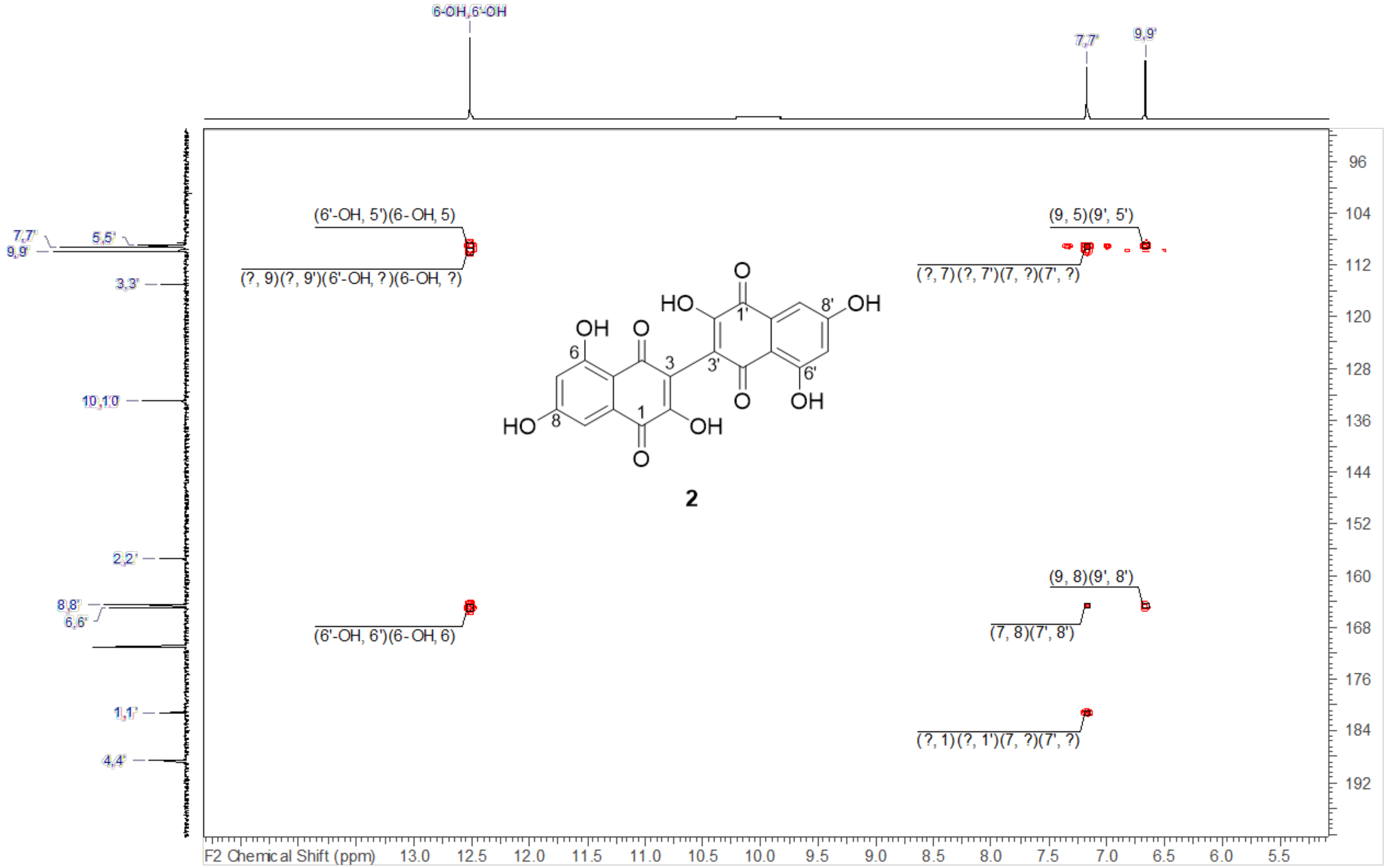

Figure S15: $^{1}H/^{13}C$ HMBC spectrum (500 MHz, acetone-d_6) of 3,3′-biflaviolin (**1**) (CNST 13 = 6 Hz)

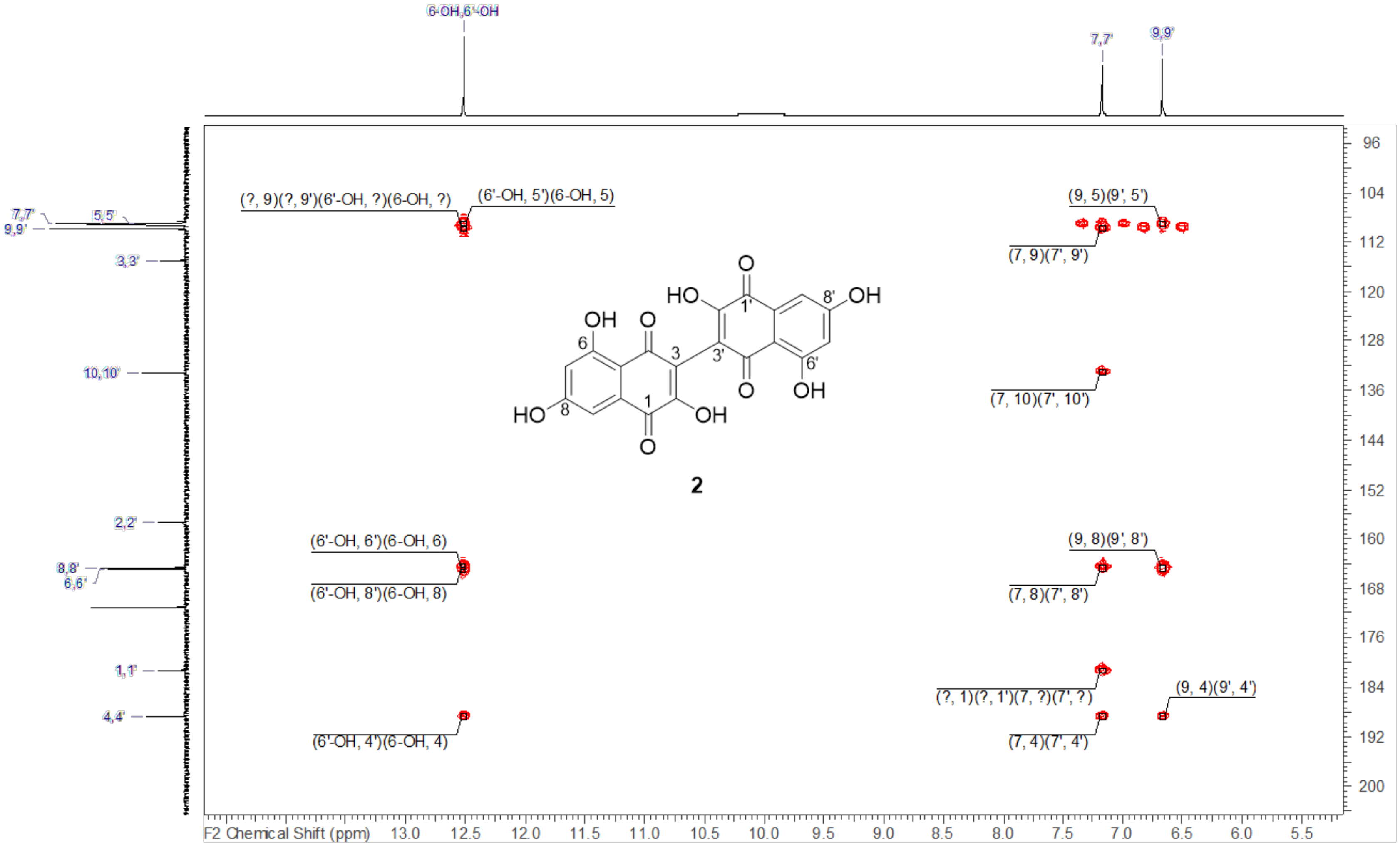

Figure S16: $^{1}H/^{13}C$ HMBC spectrum (500 MHz, acetone-d_6) of 3,3′-biflaviolin (**1**) (CNST 13 = 2 Hz).

Table S3: Flanking Positions used for phylogenetic inference as determined by Gblocks and selected evolutionary models

Table S3: Flanking positions of MAFFT alignments derived from ITS, LSU, RPB2 and TUB2 loci, which have been rated as informative characters by Gblocks and were included for the phylogenetic analysis, as well as the selected rate models by ModelFinder using Bayesian-Information Criterion (BIC).

DNA locus	Flanking positions	Proportion of the original positions	Selected Model by BIC criterion
ITS	[63-67], [103-115], [117-121], [126-131], [496-508], [520-527], [581-591], [594-598], [602-767], [774-782], [784-798], [800-807], [849-865], [867-881], [883-889], [891-904], [912-921], [946-952], [954-966]	347/1067 (32%)	SYM+I+G4
LSU	[254-459], [461-546], [548-587], [589-609], [612-664], [669-681], [683-702], [704-726], [735-861], [869-1046], [1142-1156], [1722-1767], [1770-2018], [2407-2442]	1113/2573 (43%)	K2P+R3
RPB2	[1537-1544], [1549-1556], [1558-1995], [2019-2032], [2052-2247], [2254-2296], [2300-2312], [2319-2413]	815/4054 (20%)	TIM3+F+R5
TUB2	[536-540], [547-551], [713-719], [721-730], [750-784], [857-868], [873-879], [881-908], [978-1026], [1049-1055], [1126-1190], [1305-1315], [1320-1325], [1327-2126]	1047/2624 (39%)	TIM3+F+R4

MAFFT Alignments of ITS, LSU, RPB2, TUB2 gene regions

MAFFT alignments of the four gene loci ITS, LSU, RPB2 and TUB2 of the conducted molecular phylogenetic analysis are attached separately as `.fasta` files.
